# From retina to heart: explainable machine learning using OCT and Clinical covariates for heart failure screening

**DOI:** 10.1186/s13040-026-00529-1

**Published:** 2026-02-28

**Authors:** Sona M. Al Younis, Samit Kumar Ghosh, Feryal A. Alskafi, Siamak Yousefi, Namareq Widatalla, Ahsan H. Khandoker

**Affiliations:** 1 Department of Biomedical Engineering and Biotechnology, Healthcare Engineering Innovation Group (HEIG), Khalifa University, Abu Dhabi, United Arab Emirates; 2 Department of Ophthalmology, Bascom Palmer Eye Institute, University of Miami, Miami, FL USA; 3 Department of Electrical and Computer Engineering, University of Miami, Miami, FL USA

**Keywords:** Heart failure, Retinal optical coherence tomography, Macular thickness, HF classification, Explainable machine learning, Nested loop, Machine and deep learning

## Abstract

Heart failure affects over 64 million individuals globally, contributing to elevated mortality rates and substantial healthcare costs. This study investigates the potential of retinal optical coherence tomography features combined with routine clinical variables as biomarkers for the detection of heart failure, exploring a potential avenue for improved risk assessment and screening support using explainable machine-learning tools. A comprehensive dataset of normal and heart failure patients’ demographic and medical records including retinal measurements from both eyes was used. Among the machine learning models employed, the Extreme Gradient Boosting model demonstrated the best performance, achieving an accuracy of 73.31%, a precision of 71.81%, and an area under the receiver operating characteristic curve of 0.837. Explainability analyses further revealed that macular thickness metrics, particularly in the inner temporal subfield, inner nasal subfield, and outer superior subfields of the left eye, along with key clinical indicators such as age, body mass index, and glycated hemoglobin, were the most influential predictors of heart failure status. Local explanation methods also provided patient-level reasoning consistent with overall cohort patterns. To our knowledge, this is the first study to use an integrated, explainable approach incorporating bilateral retinal optical coherence tomography measurements with routine clinical indicators for heart failure detection, providing an interpretable and accessible alternative to black-box models while helping address the cost, invasiveness, and limited accessibility of existing heart failure diagnostic tools.

**Supplementary information:**

The online version contains supplementary material available at 10.1186/s13040-026-00529-1.

## Introduction

Heart failure (HF) is a critical global health concern, affecting over 64 million individuals worldwide. It is a primary cause of hospitalizations, particularly among older adults, affecting up to 10% of people aged 75 years and beyond [1]. Currently, several types of HF are known. The most common type of HF is the left ventricular heart failure (LVHF), often known as left-sided HF, and it is believed to happen due to stiffening of the LV [2]. Congestive HF (CHF) is another type of HF that may lead to the buildup of fluid due to the impaired function of the heart. CHF can affect the left or right side of the heart, but it is most commonly associated with biventricular HF [3]. Unspecified HF (UHF) is a broad classification used when a patient has symptoms of HF, but the specific type, whether systolic, diastolic, left-sided, or right-sided, has not yet been identified. This category is often used during the initial stages of diagnosis when further testing is necessary to determine the precise nature of the HF [4, 5]. HF is diagnosed through a combination of symptom assessment, physical examination, and diagnostic testing. Blood biomarkers such as B-type natriuretic peptide (BNP) and N-terminal pro-B-type natriuretic peptide may help differentiate HF from other causes of similar symptoms. Cardiac computed tomography (CT), magnetic resonance imaging (MRI), invasive coronary angiography, and echocardiography are commonly used to evaluate heart failure related findings such as rhythm abnormalities, myocardial ischemia, and ventricular hypertrophy [6–8]. However, current clinical tools for HF assessment are often costly, resource-intensive, and limited in accessibility, particularly in low-resource or large-scale screening settings. Several modalities also carry risks associated with radiation exposure or procedural invasion. These limitations highlight the need for accurate, scalable, and non-invasive alternatives for early heart-failure detection.

The heart plays an important role in the body, and its dysfunction may affect other organs [9–12], a healthy heart is important for maintaining proper vascular function. Proper vascular function is important for the proper delivery of fluids and nutrients to the body’s organs [13]. Vascular function can be assessed by assessing retinal vasculature function. The retinal vasculature has embryological, physiological, and anatomical similarities to the coronary vasculature, and impairment in retinal microvasculature may indicate chronic vascular damage due to multiple cardiovascular risk factors, including aging, diabetes mellitus, hypertension, and conditions such as vascular dementia. These alterations likely stem from the critical role of microcirculation in regulating vascular tone to meet the oxygen demands of local tissues [14].

Retinal vasculature can be assessed by different non-invasive imaging methods such as color fundus photography, optical coherence tomography (OCT), and optical coherence tomography angiography (OCT-A). From these methods, different parameters can be captured such as arteriovenous ratio, macular total retinal thickness and many others [15].

Currently, with the advancement of artificial intelligence (AI) techniques, the development of automated image detection techniques for clinical diagnosis is feasible [4, 16]. Applying DL models for image analysis can extract meaningful features (e.g vessel geometry) from the ocular images (e.g. OCT), for prediction of diseases [17]. Vaghefi E. et al. [18] developed a DL model that can identify subjects with a high-risk score for atherosclerotic cardiovascular disease (ASCVD) from retinal images and demographics. Features extracted from retinal images, along with demographics (e.g age, race/ethnicity, and sex at birth) were combined to create the DL model, designed to predict an individual’s 10-year ASCVD risk score. Tapp. R et al. demonstrated that measurements of retinal characteristics such as the retinal arteriolar diameter, are indicative of indicators of the risk of stroke and other cardiovascular diseases [19]. Another study by J Weerts et al. demonstrated that potential differences in retinal microvascular and structural parameters (i.e., capillary vessel density and retinal layer thickness) between patients with preserved HF and control individuals exist [20].

Despite progress in fundus-based deep learning for cardiovascular risk and the emergence of retinal foundation models, few studies have evaluated how anatomically explicit OCT subfields and routine clinical factors jointly relate to HF status, with robust uncertainty reporting and patient-level explanations.

This study provides novel evidence that non-invasive OCT imaging, integrated with routine clinical covariates, can support early heart failure detection within an explainable machine-learning framework. Unlike existing diagnostic tools such as BNP testing, or echocardiography, which require specialized equipment, laboratory analysis, or trained interpretation, OCT is fast, widely available in ophthalmic settings, and does not involve radiation, contrast agents, or invasive risk. Furthermore, by leveraging anatomically explicit macular subfields and explainability algorithms (SHapley Additive exPlanations (SHAP) analysis and Local Interpretable Model-agnostic Explanations (LIME)), our approach provides clinically interpretable insights into the retinal microstructural changes associated with heart failure, offering advantages over existing black-box imaging models and enhancing its translational potential as an accessible screening tool. To summarize, the specific contributions of this study are as follows:

1. Introduces the first explainable OCT–clinical framework for heart failure detection, integrating bilateral OCT subfield measurements with routine clinical indicators to address limitations of prior methodologies.
2. Demonstrates that coupling non-invasive OCT imaging with standard clinical covariates enables early identification of heart failure, providing a practical and accessible complement to resource-intensive diagnostic methods.
3. Develops and evaluates multiple machine-learning pipelines using stratified nested cross-validation, widely used classifiers such as Decision Trees, Random Forest, Logistic Regression, Artificial Neural Networks, and XGBoost.
4. Incorporates SHAP and LIME to provide both global and patient-level interpretability, revealing anatomically grounded retinal signatures and clinically meaningful risk factors that enhance transparency and clinical trust.
5. Offers a comprehensive comparison to related work, illustrating how this multimodal, interpretable approach advances beyond earlier retinal, clinical, and deep learning based models for cardiovascular risk and HF detection.”

## Materials and methods

### Dataset and patients’ enrollment

The UK Biobank is a large, multisite, prospective cohort study comprising 502,656 UK residents aged 40–69 years who were registered with the National Health Service (NHS) in England, Scotland, and Wales. Between January 2006 and October 2010, participants attended one of 22 assessment centers, where extensive baseline data were collected through touchscreen questionnaires, verbal interviews, and physical measurements. The information obtained included sociodemographic characteristics, family history, early-life exposures, psychosocial and environmental factors, lifestyle behaviors, health status, hearing thresholds, and cognitive function, as well as self-reported medical conditions. In addition to baseline assessments, web-based questionnaires were administered to gather further information on dietary intake, occupational history, cognitive function, mental health, and gastrointestinal symptoms [21–23]. Health outcomes were tracked longitudinally through linkage to participants’ electronic health records, and disease diagnoses were coded using the International Classification of Diseases, Tenth Revision (ICD-10). Specifically, heart failure cases were identified using ICD-10 codes I50.0 (congestive heart failure), I50.1 (left ventricular failure), and I50.9 (unspecified heart failure), extracted from hospital inpatient data (UK Biobank data field 41,270). The dataset initially included a cohort of 57,636 participants.

The UK Biobank operates under a Research Tissue Bank (RTB) approval granted by the North West Multi-Centre Research Ethics Committee (MREC). This overarching approval covers all research conducted within the UK Biobank framework and permits analyses using de-identified participant data without requiring separate local ethics approval, provided that all research complies with UK Biobank’s established terms and conditions. The present study was conducted under UK Biobank application number 89,676, in accordance with the Material Transfer Agreement (MTA) and UK Biobank’s terms of access. Therefore, no additional ethics approval was required beyond the RTB approval. The study adhered to the principles of the Declaration of Helsinki, and all participants provided written informed consent. Further information about the UK Biobank’s Ethics and Governance Framework is available at https://www.ukbiobank.ac.uk/learn-more-about-uk-biobank/about-us/ethics, and details of the study design and protocols can be found on the UK Biobank website (http://www.ukbiobank.ac.uk).

**Fig. 1:**
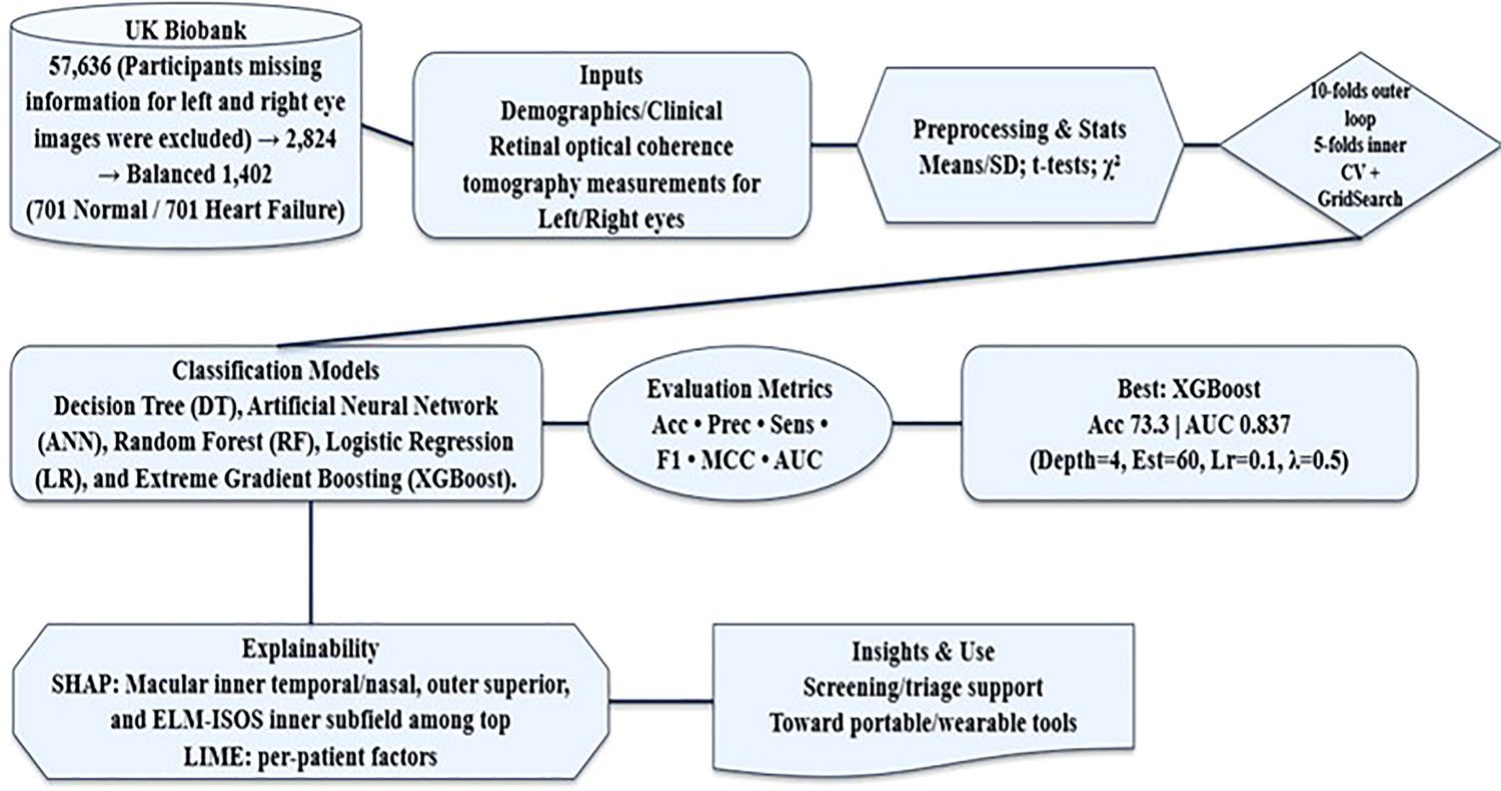
Workflow of the study, retinal OCT & clinical data to explainable ML (HF)

However, participants missing information for left and right eye images were excluded from further analysis. Consequently, the dataset comprised a total of 2824 patients, categorized into 701 Normal (participants not having any recorded disease and having right/left OCT data), 2123 HF (i.e., 744 LVHF, 701 CHF, and 678 UHF) as described in Fig. 1 To ensure a balanced study, we randomly selected 701 HF patients from the dataset, resulting in 1402 participants. The patient cohort consisted of 650 females and 752 males (age: 40 to 70 years). The dataset comprises demographic and clinical records as described in Supplement Table S2, detailing clinical, body, and lifestyle information.

### Ophthalmic assessments and data preprocessing

Ophthalmic assessments were conducted for a subset of participants between June 2009 and July 2010 at six assessment centers. These assessments included visual acuity (LogMAR) measurements, refractive error, intraocular pressure (IOP), and ophthalmic imaging captures. Baseline best-corrected visual acuity was measured using a computerized semi-automated system at a distance of 3 meters. Autorefraction was performed using an RC5000 Auto Refkeratometer (Tomey, Nagoya, Japan), and the spherical equivalent was calculated by summing the spherical power and half of the cylindrical power. Corneal compensated intraocular pressure (IOPcc) was measured with the Ocular Response Analyzer (ORA; Reichert Corp., Philadelphia, PA), with one measurement taken per eye. Participants with possible eye infections or recent eye surgery (within four weeks) were excluded from IOP measurements. Single-field color fundus photographs (45° field-of-view, centered on the optic disc and macula and including both) and macular OCT scans were captured using a digital Topcon-1000 integrated ophthalmic camera (Topcon 3D OCT1000 Mark II, Topcon Corp., Tokyo, Japan) [24]. This was carried out following the collection of intraocular pressure, autorefraction, and visual acuity values. Using the 3-dimensional 6 × 6 mm macular volume scan mode, which consists of 128 horizontal B-scans in a raster pattern and 512 A-scans per B-scan, the OCT scans were taken in mesopic lighting without pupillary dilation. First to be taken was the right eye, then the left [25]

The retinal measurements extracted from the OCT imaging included External Limiting Membrane (ELM) and Inner Segment-Outer Segment (ISOS) thickness (average, central subfield, inner subfield, and outer subfield), Inner Nuclear Layer (INL) and ELM thickness (average, central subfield, inner subfield, and outer subfield), INL- Retinal Pigment Epithelium (RPE) thickness (average, central subfield, inner subfield, and outer subfield), Inner Segment-Outer Segment (ISOS) junction-RPE thickness (average, central subfield, inner subfield, and outer subfield), Macular thickness (average, central subfield, inner subfield, and outer subfield), Intraocular Pressure (IOP), IOP Corneal Compensated, and IOP Goldmann Correlated. Detailed descriptions and definitions of these retinal optical features are provided in Supplement Table S1. Alongside the systemic health indicators, the retinal OCT measurements of the left and right eyes were included, to enhance the understanding of retinal changes in HF patients as described in Supplement Table S3 and Supplement Table S4, offering a comprehensive view of retinal features.

The patient cohort was categorized into two groups: Normal, and Heart Failure (Left Ventricular Heart Failure, Congestive Heart Failure, and Unspecified Heart Failure). The statistical analysis involved computing each parameter’s means, standard deviations, and counts. We used an independent two-sample t-test to evaluate whether there were significant differences between the two groups. The t-test assumes unequal variances, providing robust comparisons between the two populations. For categorical variables, such as the gender feature, we used a chi-square test of independence to determine whether the distribution of the categories (e.g., Male and Female) differed significantly between the two groups. For each feature, the resulting smaller *p*-value (e.g., less than 0.05) indicates that the feature’s distribution differs significantly between the normal and HF samples. Additionally, box plots were generated for each demographic/clinical and retinal measurement to visually illustrate the distribution of data points across the normal and heart failure categories. Data processing and analysis were performed using Python. The pandas library was used for data management and preprocessing, and SciPy was applied for statistical analyses. Data visualization, including box plots, was carried out using Seaborn and Matplotlib. The processed data were then used as input for the classification models

### Proposed classification models

To compare the effectiveness of various ML models, five models were implemented for the classification of HF patients [26], including Decision Tree (DT) [27], Artificial Neural Network (ANN) [28], Random Forest (RF) [29], Logistic Regression (LR) [30], and Extreme Gradient Boosting (XGBoost) [31]. All models were implemented as scikit-learn Pipelines to prevent data leakage. Numeric features were imputed using the median, while categorical features were represented using one-hot encoding with handle_unknown=‘ignore’. A summary table listing all variables used in the final classification models (demographic and clinical characteristics, and bilateral OCT features), including their names, units, proportions of missing data in the raw extracted variables, and the corresponding preprocessing strategy (numeric imputation or categorical encoding), is provided in Supplement Table S5. Numeric features were standardized using z-score normalization for Logistic Regression and ANN models to ensure stable gradient-based optimization, whereas tree-based models (Decision Tree, Random Forest, and XGBoost) were trained on unscaled inputs due to their scale-invariant splitting mechanisms. We performed stratified nested cross-validation on the full cohort (*N* = 1,402). For each model, hyperparameters were selected in an inner 5-fold CV using GridSearchCV (scoring: accuracy) after fitting all preprocessing steps within the pipeline to prevent leakage [32]. These hyperparameter values were selected based on a combination of domain expertise and experimentation, and Table 1 contains all the tuned parameters for the ML models [26, 33]. The outer loop used stratified K-fold CV (e.g., K = 10) to estimate generalization: on each outer test fold we computed Accuracy, Precision, Sensitivity (Recall), F1, MCC, Matthews Correlation Coefficient (MCC), *p*-value, and Area Under the Receiver Operating Characteristic Curve (ROC AUC), and then reported the mean ±95% CI (confidence interval) across outer folds (non-parametric percentile CIs). Furthermore, the SHapley Additive exPlanations (SHAP) [34]analysis and the Local Interpretable Model-agnostic Explanations (LIME) algorithm [35]were applied on representative outer-fold test cases to enhance the global and local interpretability of models, utilizing OCT and medical features for the prediction process in the XGBoost model (proven to provide best results), providing valuable insights into the model’s decision-making process. The final hyperparameter settings for each model are presented in Table 1. All experiments were completed on a single NVIDIA GeForce M×350 (2 GB) GPU with an Intel Core i5-1135G7 (11th Gen) CPU.

**Table 1:** Classification models and hyperparameters tuning

Classification Model	Hyperparameters	Best Combination
DT	max depth: [3, 5, 7, 10], min samples leaf: [10, 20, 50, 100], criterion: [‘gini’, ‘entropy’], splitter: [‘best’, ‘random’]	max depth: 5, min samples leaf: 100, criterion: ‘entropy’, splitter: ‘best’.
ANN	hidden layer sizes: [50, 100], activation: [‘logistic’, ‘relu’], solver: [‘lbfgs’, ‘adam’], alpha: [0.0001, 0.01, 0.1]	hidden layer sizes: 100, activation: ‘logistic’, solver: ‘adam’ alpha: 0.1,
RF	max depth: [10, 20, 30], min samples leaf: [1, 14], min samples split: [5, 12], estimators: [100, 200], criterion: [‘gini’, ‘entropy’]	max depth: 10, min samples leaf: 1, min samples split: 5, estimators: 100, criterion: ‘entropy’,
LR	solver: [‘liblinear’, ‘lbfgs’], penalty: [‘l2’] C: [0.1, 1.0, 10.0], max iter: [100, 500, 1000]	solver: ‘liblinear’, penalty: ‘l2’, C: 10.0, max iter: 100
XGBoost	max depth: [4, 5], estimators: [60], learning rate: [0.01, 0.1], subsample: [1.0], colsample bytree: [0.5, 1.0], reg lambda: [0.5, 1.0]	max depth: 4, estimators: 60, learning rate: 0.1, subsample: 1.0, colsample bytree: 0.5, reg lambda: 0.5

## Results

**Fig. 2:**
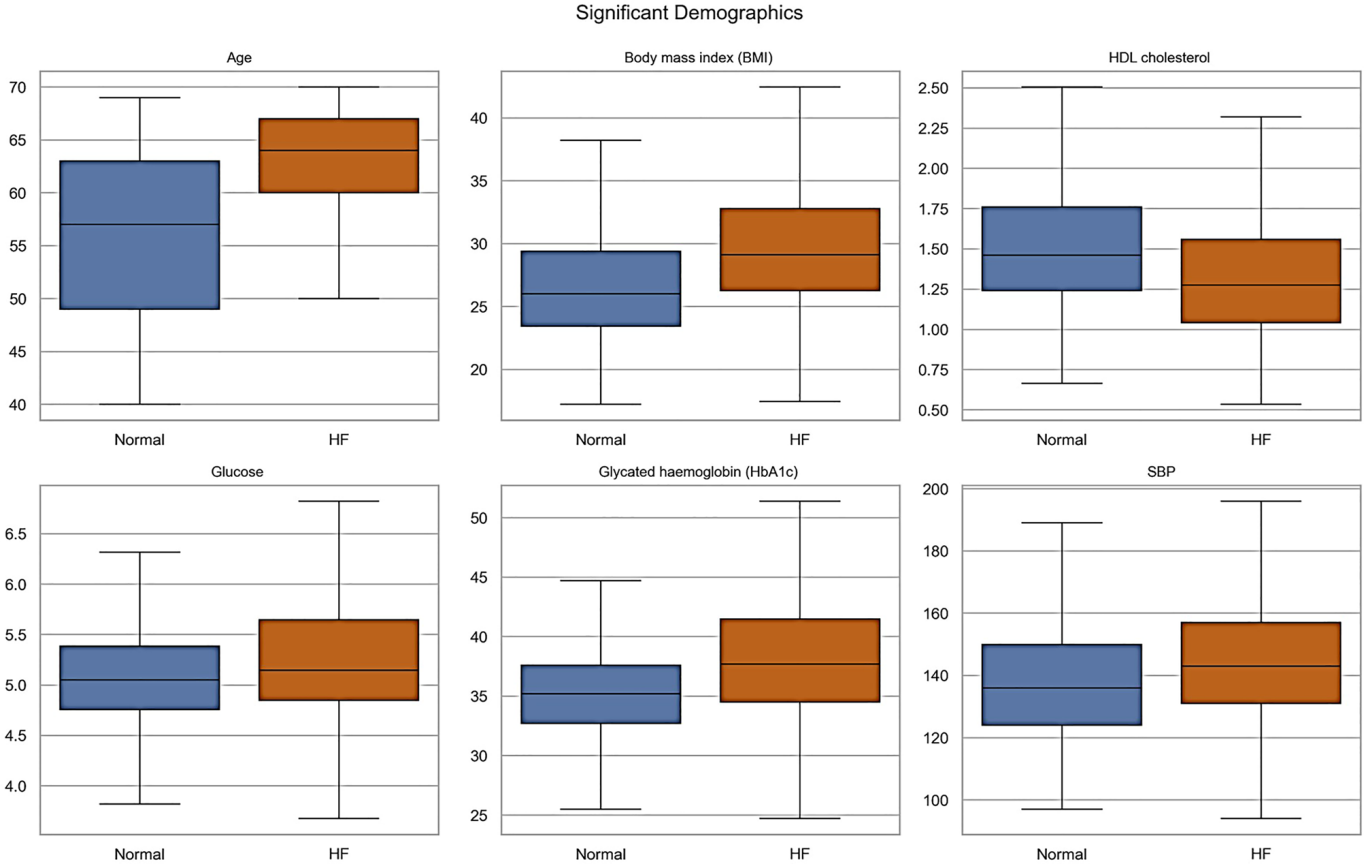
Box plots of the continuous demographic and clinical characteristics found to be significantly different between the normal and heart failure groups

**Fig. 3:**
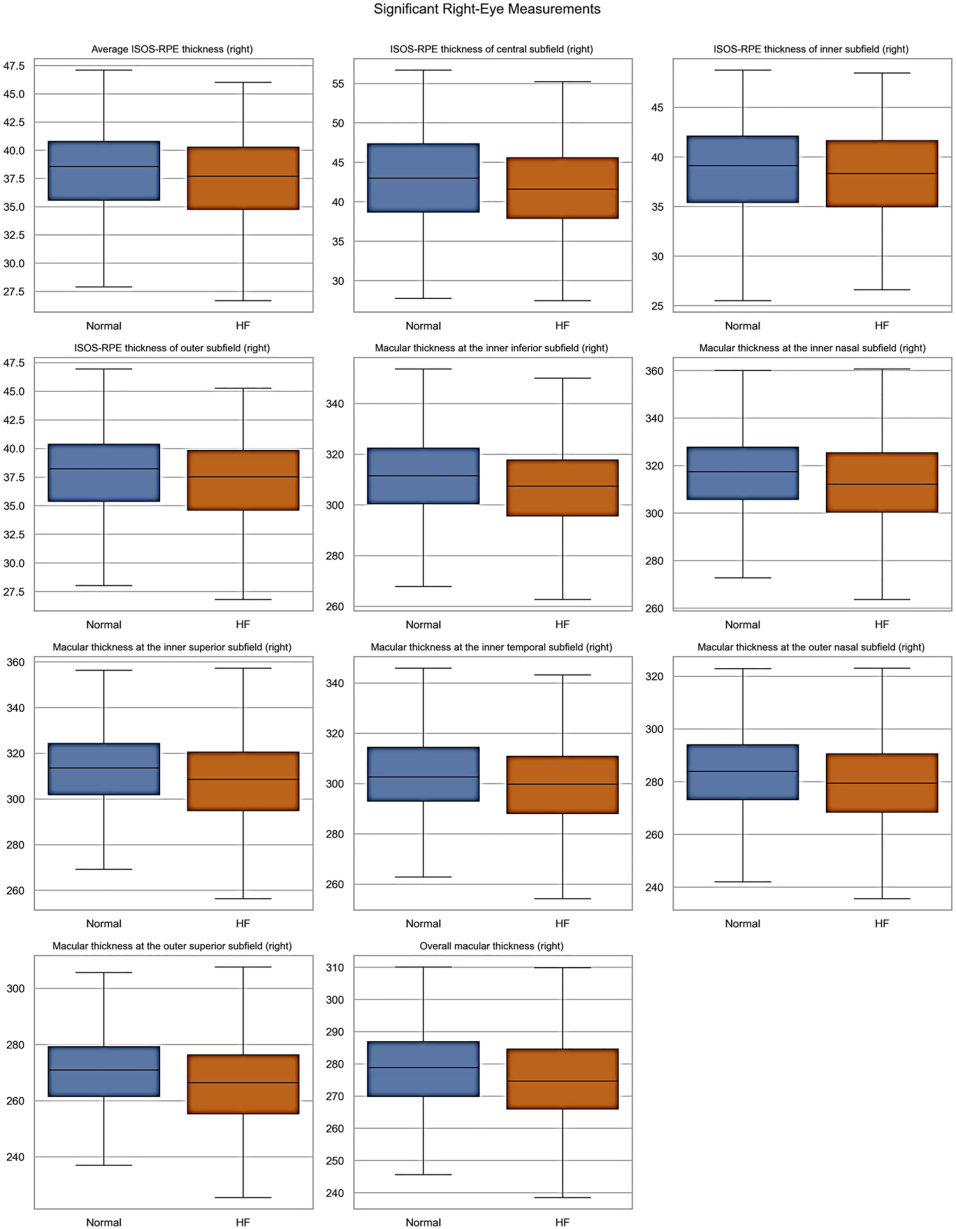
Box plots of significant left eye parameters by heart failure type

**Fig. 4:**
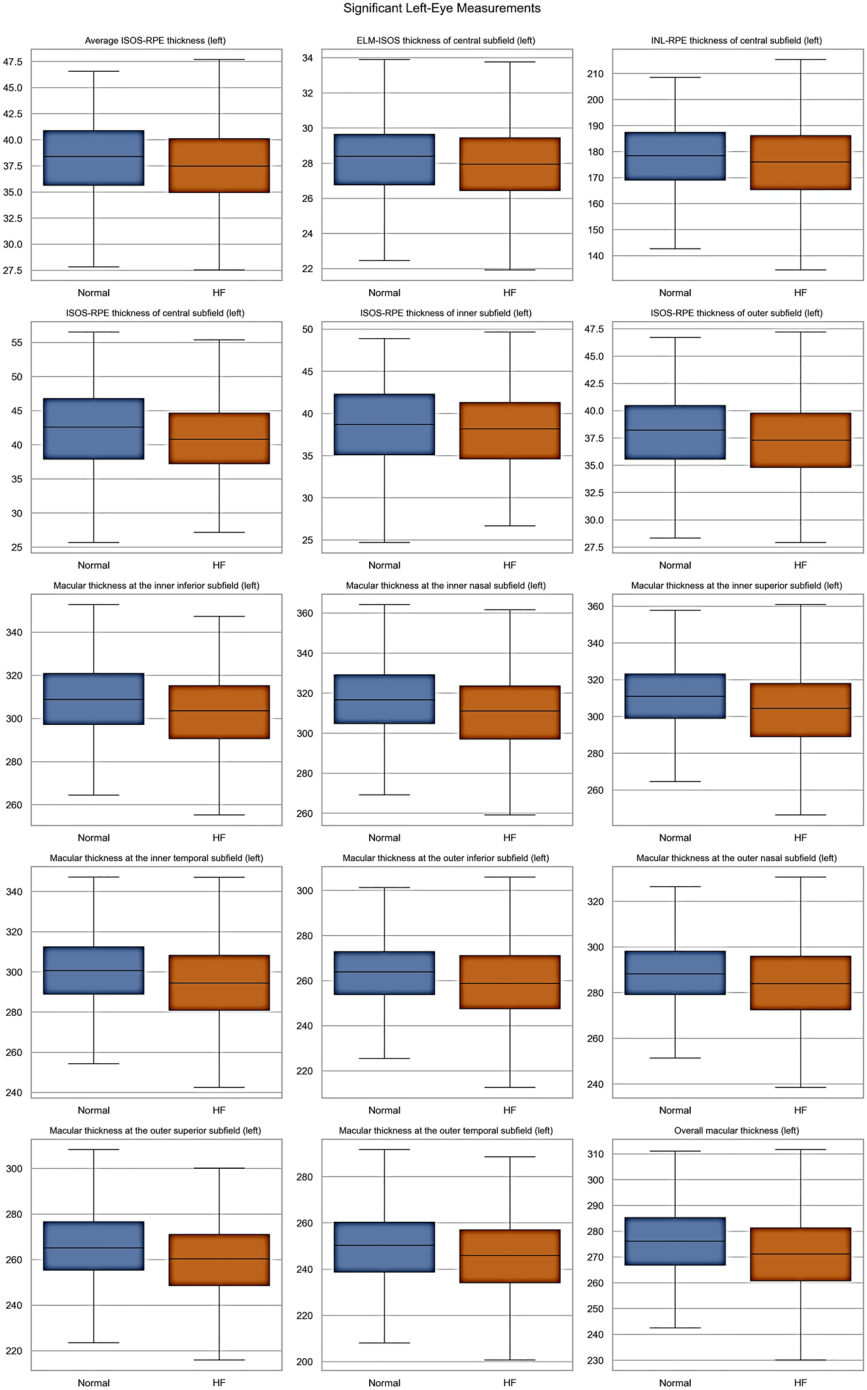
Box plots of right eye parameters by heart failure type

All demographic and clinical characteristics were found to be statistically significant between the Normal and Heart Failure groups, except for the diastolic blood pressure as reported in Supplement Table S2. The distribution of these continuous demographic and clinical characteristics is further shown in Fig. 2 Retinal OCT measurements for the left and right eyes were statistically analyzed and reported Supplement Table S2, and S3, respectively. To visually highlight the spread and variability of retinal parameters across the normal and HF groups, statistically significant retinal OCT measurements are shown in Figs. 3, and 4, displaying the median, interquartile ranges, and any outliers for the left and right eyes, respectively.

For each approach, reliable, low-variance configurations were obtained using grid searches over small, literature-motivated ranges (Table 1). To balance bias and variance on mixed clinical/OCT inputs, decision tree recommended a shallow, regularized structure entropy, max depth = 5, minimum leaf = 100, and splitter = best. With logistic activation, Adam, and α = 0.1, the ANN chose a compact 1 × 100 hidden layer, suggesting that greater weight decay enhanced generalization in the 80/20 split. At random forest with max depth = 10, min samples split = 5, min leaf = 1, and 100 estimators, converged to entropy, meeting the one hot encoding data size and feature sparsity. Logistics with L2, C = 10, solver = liblinear, regression performed best, which is consistent with mild regularization on standardized numeric. With max depth = 4, estimators = 60, learning rate = 0.1, subsample = 1.0, colsample bytree = 0.5, and reg lambda = 0.5, XGBoost achieved the strongest overall configuration. This combination prevents overfitting by limiting tree depth and encouraging feature subsampling, while maintaining discriminative interactions between OCT subfields and clinical covariates.

**Fig. 5:**
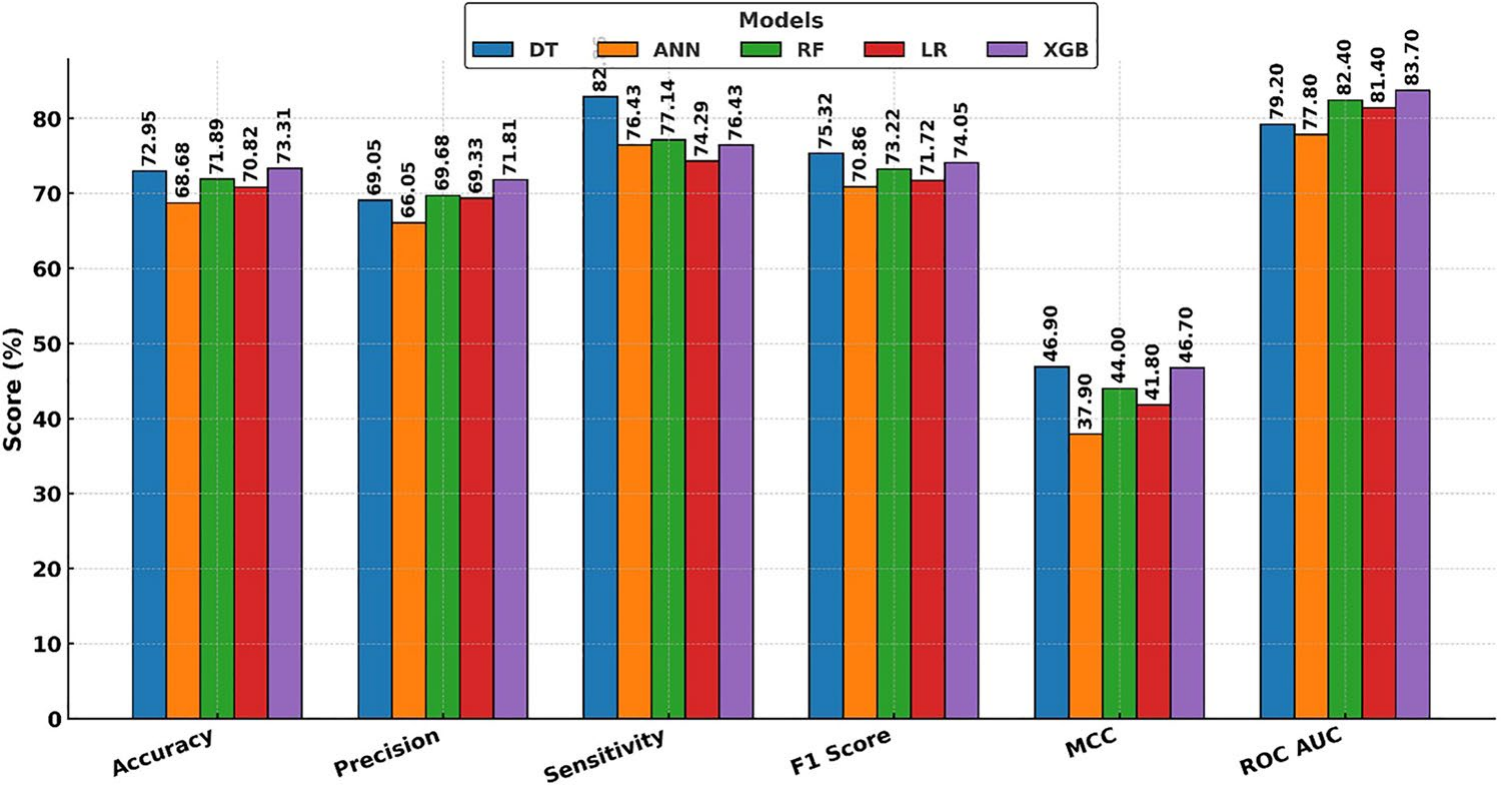
Comparative performance of five classifiers on normal vs. Heart failure classification

**Fig. 6:**
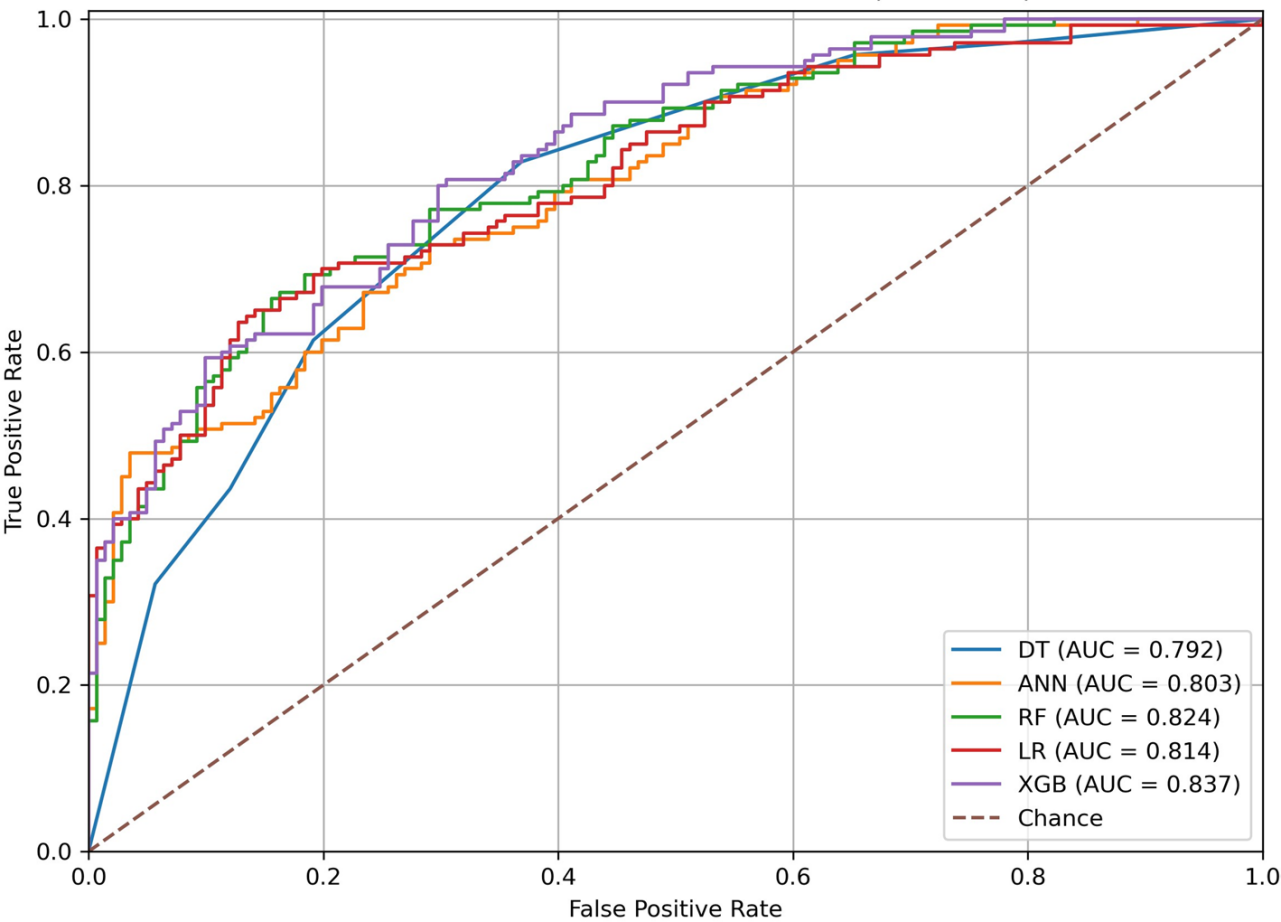
A visualization of the ROC curves for the five classification models

Across models, the test-set performance indicated modest discrimination between normal and HF groups, with ROCAUC values consistently exceeding chance levels. As reported in Table 2, XGBoost achieved the highest discrimination (ROC AUC = 0.837; 95% CI 0.790–0.879), with balanced operating characteristics accuracy = 73.31% [68.33–78.29], precision = 71.81% [64.47–78.91], sensitivity = 76.43% [69.50–83.45], F1 = 74.05% [68.18–79.23], and MCC = 0.467 [0.365–0.564]). Random Forest and Logistic Regression followed closely (AUC = 0.824 and 0.814, respectively), while Decision Tree and ANN trailed but remained robust (AUC = 0.792 and 0.778). Notably, all models showed *p* = 0.001 for AUC > 0.5, confirming acceptable separability of classes on the held-out test set. The combination of OCT macular subfield metrics with demographic/clinical variables supports moderate discrimination of HF status in this cohort. The stronger MCCs for XGBoost/RF indicate better balance across true-positive and true-negative rates, and the tight bootstrap intervals suggest stable generalization in this 80/20 framework. Grouped bars in Fig. 5 summarize the classification models performance on the 20% held out test set, and Fig. 6 represents the ROC curves for the five classification models. The visual pattern shows XGBoost with the best overall discrimination (highest AUC) and balanced accuracy/F1; DT emphasizes sensitivity at the expense of precision; RF and LR perform closely and consistently across metrics, while ANN is comparatively weaker. These findings motivated using SHAP/LIME on the XGBoost model to characterize which retinal and clinical features drove predictions at cohort and patient levels.

**Table 2:** Classifier performance on the held-out test set (normal vs. Heart failure)

Model	Accuracy (%)	Precision (%)	Sensitivity (%)	F1 Score (%)	MCC	p-value	ROC AUC
DT	72.95 (67.97–77.94)	69.05 (61.85–76.07)	82.86 (76.35–89.51)	75.32 (69.86–80.51)	0.469 (0.365–0.565)	0.001	0.792 (0.740–0.841)
ANN	68.68 (63.35–74.02)	66.05 (58.79–73.25)	76.43 (69.59–83.33)	70.86 (65.23–76.45)	0.379 (0.275–0.486)	0.001	0.803 (0.767–0.844)
RF	71.89 (66.54–77.22)	69.68 (62.25–77.16)	77.14 (70.42–84.03)	73.22 (67.35–78.51)	0.440 (0.332–0.542)	0.001	0.824 (0.776–0.869)
LR	70.82 (65.48–76.51)	69.33 (62.00–76.58)	74.29 (67.33–81.56)	71.72 (65.53–77.46)	0.418 (0.308–0.529)	0.001	0.814 (0.765–0.864)
XGBoost	**73.31 (68.33–78.29)**	**71.81 (64.47–78.91)**	**76.43 (69.50–83.45)**	**74.05 (68.18–79.23)**	**0.467 (0.365–0.564)**	**0.001**	**0.837 (0.790–0.879)**

Accuracy, Precision, Sensitivity, F1, MCC, and ROC AUC with 95% bootstrap CIs; one-sided permutation *p*-values for AUC > 0.5

**Fig. 7:**
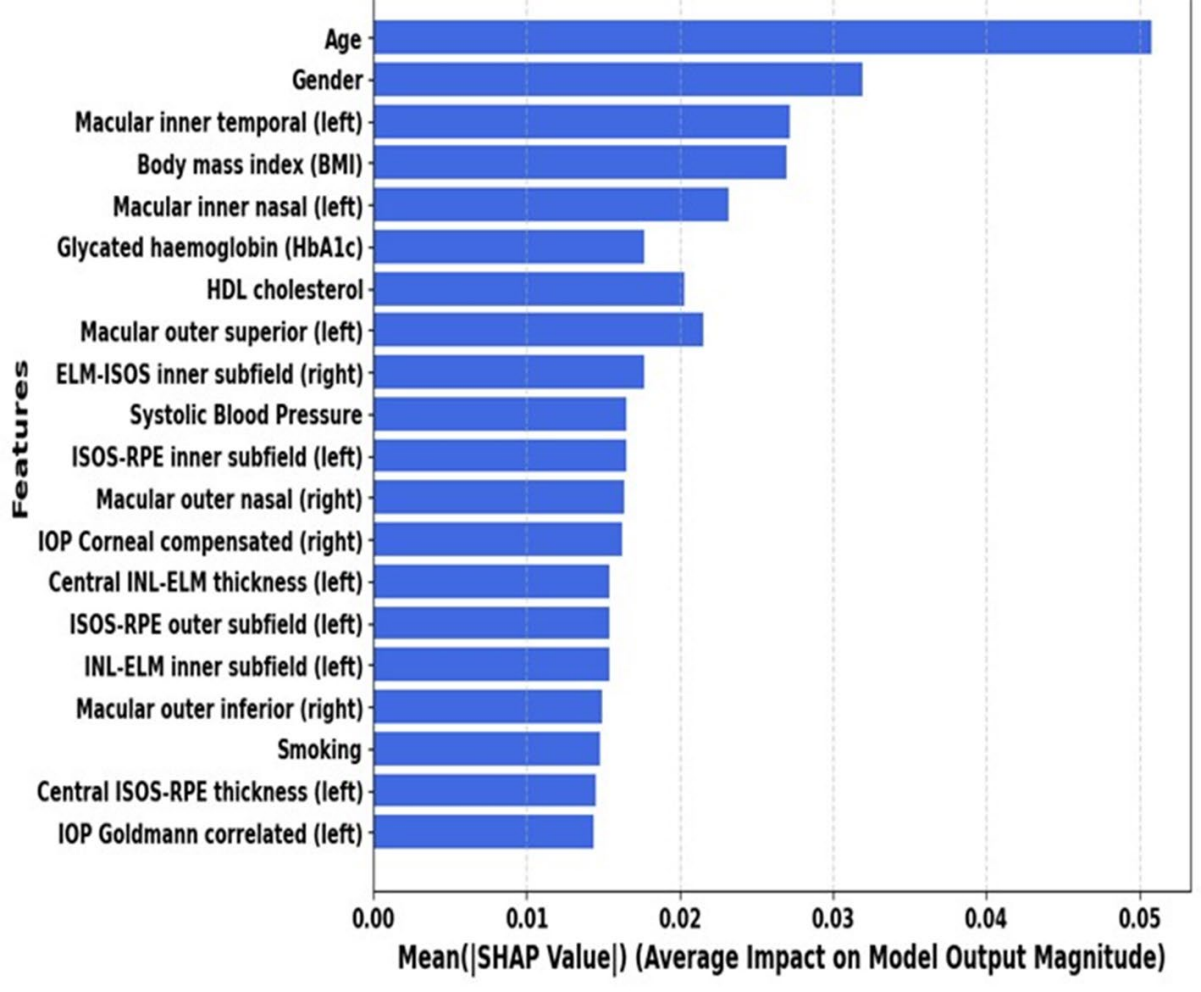
Ranking of top 20 feature importance by SHAP algorithm for predicting HF in the XGBoost, utilizing medical records and OCT features

By identifying which retinal subfields and clinical markers drive risk and in which direction, these explainability tools are essential in this context. They also allow clinicians to explore whether model patterns align with known mechanisms and to detect potential spurious associations, which may support interpretability and quality assessment of the model outputs. The SHAP analysis applied to the XGBoost model provides a transparent view of the features contributing to HF classification, helping to clarify which retinal and clinical variables influence the model’s decisions. Figure 7 shows the top 20 SHAP values, herein, Age, gender, and BMI remain central to HF classification, reflecting their fundamental roles in cardiovascular health. Several macular thickness measurements from the left eye’s inner subfields (temporal, nasal, and outer superior regions) ranked prominently among the top features. The inclusion of right eye measurements (e.g., ELM-ISOS inner and macular outer nasal) further highlights the systemic nature of HF related retinal changes and the bilateral relevance of retinal metrics. Parameters such as IOP, ISOS-RPE, and INL-ELM thicknesses were also identified as significant contributors. However, retinal features, especially macular thickness, correlate with HF through microvascular dysfunction, neurovascular imbalance, and systemic inflammation. These changes reflect HF-related vascular stress, edema, and hypoxia, suggesting a link between ocular biomarkers and systemic cardiovascular changes, warranting further investigation. Glycated hemoglobin (HbA1c) is also ranked high, reflecting the significant role of metabolic dysfunctions, such as diabetes, in HF pathophysiology. HDL cholesterol and systolic blood pressure, both critical cardiovascular health indicators, ranked moderately in importance. This aligns with their established influence on cardiac workload and vascular health, which are directly linked to heart failure development.

**Fig. 8:**
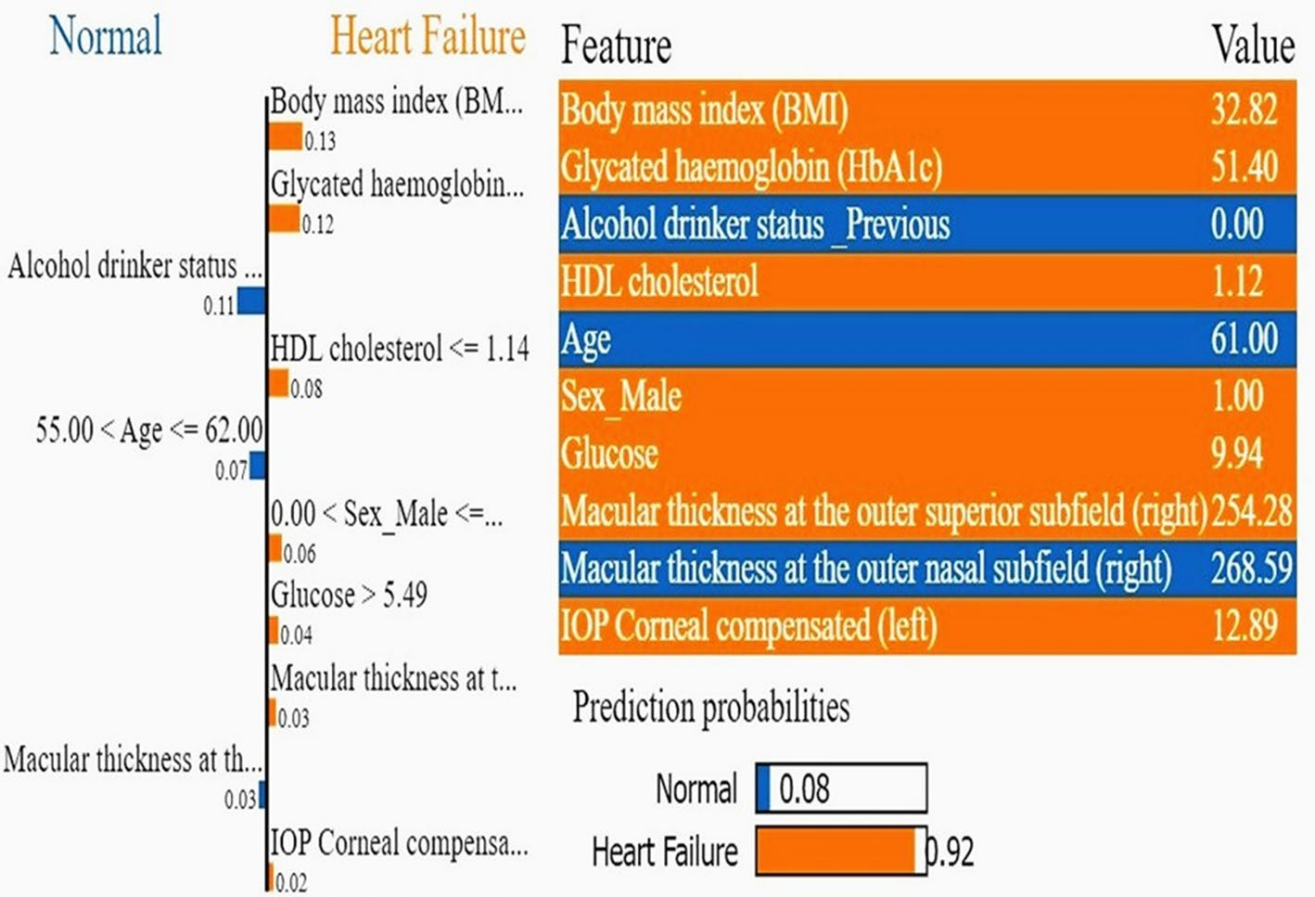
A visualization of LIME model scores for patient ID 2 using the XGBoost model

Furthermore, The LIME based explanation for Patient ID2 (Fig. 8 highlights a 92% probability of HF. Notably, elevated BMI (32.82), HbA1c (51.40), and HDL cholesterol (1.12) indicate metabolic risk factors linked to HF. Retinal parameters, including macular thickness in the outer nasal (268.59 µm) and superior (254.28 µm) subfields in the right eye, and IOP corneal compensated (12.89) in the left eye further support systemic vascular involvement in HF. SHAP was used to assess global, cohort-level feature contributions, whereas LIME was applied to provide local, patient-specific explanations; the complementary roles of these methods are further discussed in the Discussion. Upon comparing the findings with the *p*-value results in Supplement material S2, S3, and S4, consistency is evident, underscoring the significance and efficacy of incorporating these variables in the detection of HF, as explained further in the discussion. Notably, our findings surpass those previously reported in the literature regarding the correlation between OCT markers and the prediction of HF (Table 5).

We performed two additional sets of experiments in which the models were trained and evaluated using retinal OCT-derived features only and non-retinal (clinical) features only, respectively. This analysis allows direct quantification of the predictive contribution of retinal imaging features compared with clinical covariates alone.

When trained using retinal OCT features only, all machine learning models demonstrated moderate discriminative performance, indicating that retinal structure contains meaningful information related to heart failure status (Table 3). Specifically, the XGBoost model achieved a AUROC of 0.75 (95% CI: 0.69–0.78), with consistent performance across accuracy, F1 score, and Matthews correlation coefficient. These results demonstrate that OCT-derived retinal features alone provide substantial predictive signal, although performance was lower than that achieved when combining retinal and clinical information.

**Table 3:** Classifier performance on the held-out test set (normal vs. Heart failure), using OCT features only

Model	Accuracy (%)	Precision (%)	Sensitivity (%)	F1 Score (%)	MCC	p-value	ROC AUC
DT	62.95 (57.97–67.94)	59.05 (51.85–66.07)	**72.86 (66.35–79.51)**	**65.32 (59.86–70.51)**	0.37 (0.27–0.47)	0.0010	0.69 (0.64–0.74)
ANN	64.02 (59.04–69.00)	**64.10 (56.91–71.35)**	63.57 (56.00–70.95)	63.84 (57.65–69.43)	0.38 (0.28–0.48)	0.0010	0.72 (0.67–0.77)
RF	63.31 (58.33–68.29)	60.44 (53.06–67.70)	70.00 (63.05–76.49)	64.92 (59.09–70.48)	0.37 (0.27–0.47)	0.0010	0.74 (0.69–0.78)
LR	63.67 (58.68–68.66)	62.00 (54.86–68.98)	67.14 (60.07–74.46)	64.48 (58.57–69.86)	0.38 (0.28–0.48)	0.0010	0.73 (0.68–0.78)
XGBoost	**64.03 (58.68–69.36)**	61.90 (54.54–68.75)	68.57 (61.43–75.32)	65.09 (59.37–70.54)	**0.38 (0.28–0.49)**	**0.0010**	**0.75 (0.69–0.78)**

Accuracy, Precision, Sensitivity, F1, MCC, and ROC AUC with 95% bootstrap CIs; one-sided permutation *p*-values for AUC >0.5

In contrast, models trained using non-retinal clinical features only showed lower performance, with AUROC values close to chance level for several classifiers (Table 4). For example, the XGBoost model achieved an AUROC of 0.63 (95% CI: 0.56–0.70), and the Decision Tree model demonstrated no statistically significant discrimination. This highlights the limited predictive capacity of clinical variables alone in this cohort. Together, these findings support our central claim that retinal OCT features contribute independently and meaningfully to heart failure prediction, and that their integration with clinical data yields the strongest overall performance.

**Table 4:** Classifier performance on the held-out test set (normal vs. Heart failure), using non-retinal clinical features only

Model	Accuracy (%)	Precision (%)	Sensitivity (%)	F1 Score (%)	MCC	p-value	ROC AUC
DT	50.18 (44.48–55.87)	50.00 (42.22–58.28)	51.43 (42.86–59.57)	50.70 (43.61–57.53)	0.004 (0.111–0.114)	0.3397	0.513 (0.443–0.582)
ANN	57.65 (52.66–62.99)	59.63 (50.42–68.47)	46.43 (38.73–54.68)	52.21 (44.73–58.92)	0.156 (0.051–0.268)	0.0140	0.578 (0.515–0.645)
RF	58.36 (52.67–64.06)	58.39 (50.00–66.14)	57.14 (49.19–66.02)	57.76 (50.95–64.54)	0.167 (0.052–0.281)	0.0020	0.632 (0.563–0.697)
LR	58.72 (53.38–64.06)	59.09 (51.20–66.90)	55.71 (47.44–64.03)	57.35 (50.20–64.06)	0.174 (0.067–0.282)	0.0010	0.598 (0.533–0.663)
XGBoost	**60.14 (54.09–66.19)**	**60.29 (51.82–68.70)**	**58.57 (50.35–67.38)**	**59.42 (52.55–66.88)**	**0.203 (0.081–0.323)**	**0.0020**	**0.633 (0.565–0.699)**

Accuracy, Precision, Sensitivity, F1, MCC, and ROC AUC with 95% bootstrap CIs; one-sided permutation *p*-values for AUC > 0.5

## Discussion

This study provides useful information regarding the importance of retinal OCT features and clinical/demographic factors in identifying patients with heart failure. Explainability analyses (SHAP/LIME) consistently identified age, BMI, glycemic status (HbA1c), and macular thickness metrics specifically, the inner temporal/nasal and outer superior subfields of the eye as significant predictors. However, XGBoost outperformed the other five benchmark models in terms of overall discrimination (test ROC AUC ≈0.84) with balanced Accuracy/F1. These results showcase the capability of incorporating retinal OCT features into ML algorithms, indicating their potential as valuable tools for screening for heart failure diagnosis and treatment.

Microvascular remodeling, metabolic stress, and systemic hemodynamic load all affect retinal structure and microvasculature. Fundamental microcirculation research demonstrates how endothelial signaling and arteriolar tone combine to provide local oxygen demand for flow pathways that are likely disrupted in heart failure by neurohormonal activation, congestion, and inflammation [14]. Retinal vascular morphology and cardiometabolic risk variables (age, blood pressure, BMI, and glycemia) are strongly correlated, according to large population studies [19].

Although this study focuses on cross-sectional discrimination against heart failure status, accumulating evidence supports the biological plausibility that retinal OCT and OCTA metrics may reflect cardiovascular dysfunction over time. OCTA studies in HFpEF and dilated cardiomyopathy have shown measurable alterations in capillary density and retinal perfusion associated with disease severity [20, 36]. Large cohort studies have further demonstrated that retinal structural and microvascular parameters, including macular thickness and vascular metrics, track long-term cardiometabolic burden and systemic risk [19, 24]. Recent reviews reinforce the potential value of OCT-based biomarkers for cardiovascular monitoring and emphasize the need for prospective, longitudinal OCT studies in heart-failure populations [37, 38]. While these findings support the rationale for exploring OCT-derived features in HF, establishing causal or temporal relationships between retinal changes and HF progression will require long-term follow-up datasets and interventional studies.

## Related work

Several studies have examined heart failure or cardiovascular risk prediction from retinal images. As shown in Table 5, in most recent study [39], ultra-large natural-image foundation models (e.g., DINOv2) have been compared against RETFound for systemic disease prediction; although generic vision models can perform competitively for ocular tasks, RETFound still yields superior AUROC values (≈0.73–0.80) for myocardial infarction, ischaemic stroke, and specifically AUROC of 0.796 for heart failure prediction [39]. This work emphasizes representation learning rather than explainable subfield-level biomarkers. Deep-learning derived retinal cardiovascular biomarkers such as Reti-CVD and Reti-CAC have also been investigated for their ability to stratify cardiovascular risk, including outcomes related to heart failure [40]. These models use convolutional neural networks applied to color fundus photographs, often in combination with traditional clinical risk scores, and have been evaluated across large cohorts in both the UK Biobank and several Asian population studies. Their reported performance for composite cardiovascular disease outcomes, which include heart failure hospitalization among other major events, typically yields C-statistics in the mid-0.6 to low-0.7 range. Moreover, Kadry et al. reported that retinal fundus images are crucial in diagnosing CVD using Chronological Chef Based Optimization Algorithm (CCBOA) and Deep Residual Network (DRN) [41]. Although these biomarkers demonstrate the feasibility of using retinal imaging for broad cardiometabolic risk profiling, they are not specifically optimized for heart-failure discrimination, and heart failure is generally incorporated as part of a composite endpoint rather than evaluated independently. Moreover, these approaches rely exclusively on fundus images and do not incorporate layer-resolved OCT features or explainability frameworks such as SHAP or LIME, which limits anatomical interpretability relative to the method proposed in the present study [37, 38]. In contrast, Zhou et al. developed RETFound [42], a retinal foundation model trained using self-supervised learning (SSL) on over one million unlabeled fundus and OCT images, and fine-tuned it for systemic disease detection, including incident heart failure. Using a large internal cohort (MEH–AlzEye) and external validation in the UK Biobank, RETFound achieved strong performance for three-year HF incidence prediction (AUROC ≈ 0.79), outperforming conventional ImageNet-pretrained and alternative SSL models. Unlike the present study, RETFound operates directly on raw retinal images and is designed for large-scale, label-efficient prediction of future HF risk rather than explainable, feature-level HF discrimination. [42].

**Table 5:** Summary table of related work

Study	Imaging modality/inputs	HF endpoint	Model type	Population/sample	Reported HF performance	Key differences vs. present study
[39]	Color fundus photographs; foundation models pre-trained on natural images vs retina-specific foundation model	Incident HF, MI, ischaemic stroke	DINOv2 (natural-image foundation model) vs RETFound	Moorfields/AlzEye with external validation on UK Biobank	RETFound AUROC for HF 0.732–0.796, consistently higher than DINOv2 range 0.663–0.771	Compares different foundation models for systemic disease from fundus images; no OCT or routine clinical variables; emphasis on representation learning rather than explainable subfield-level biomarkers.
[40]	Color fundus photographs; DL-derived retinal biomarker (Reti-CVD/Reti-CAC) combined with traditional risk scores	Composite CVD events including HF hospitalization and HF-related outcomes	CNN-based retinal biomarker plus Cox models and risk scores	UK Biobank and Asian cohorts (10,000s of participants)	C-statistics for composite CVD typically mid-0.6s to low-0.7s; HF included in outcome definition but not primary endpoint	Demonstrate feasibility of retinal DL biomarkers for broad CVD risk (including HF) but not optimized for HF-specific discrimination; inputs limited to fundus; no layer-resolved OCT or SHAP/LIME explanations.
[42]	Color fundus photographs (CFP) and OCT B-scans; RETFound foundation model pretrained with self-supervised masked autoencoder on ~1.6 million unlabelled retinal images, then fine-tuned.	3-year incident heart failure (new HF events) from linked hospital admission data (ICD-10 I50)	Vision Transformer–based self-supervised foundation model (RETFound) fine-tuned for HF prediction	Internal: MEH-AlzEye cohort (353,157 patients with retinal imaging linked to national hospital records). External: UK Biobank participants with CFP/OCT and longitudinal EHR follow-up	Internal MEH-AlzEye: AUROC ≈0.79 for 3-year HF incidence prediction from CFP; RETFound outperformed ImageNet-pretrained and other SSL baselines for both CFP and OCT, and also achieved the best HF performance in external UK Biobank evaluation	Focuses on future HF risk prediction (3-year incidence) rather than prevalent HF vs. control classification; uses raw CFP/OCT images only without explicit retinal thickness subfields or routine clinical covariates; aims to build a large, label-efficient, general retinal foundation model. In contrast, the present study uses tabular OCT macular subfield metrics plus clinical variables with SHAP/LIME for interpretable HF screening on a smaller but anatomically explicit feature set.
[26]	Macular OCT measurements (left, right, bilateral eyes)	HF phenotypes (LVHF, CHF, UHF) rather than HF vs. control.	Several ML classifiers (e.g., logistic regression) using tabular OCT features.	UK Biobank HF cohort; ~1,400 participants	Best performance for unspecified HF using bilateral OCT: accuracy 67.4%, AUC 0.692.	First to show OCT thickness alone carries HF-related signal, but without clinical covariates performance is modest; current study extends this by adding routine clinical variables and advanced explainability, achieving higher AUC (0.837) for HF vs. control.
This study	OCT measurements (bilateral eyes) and clinical covariates.	HF vs. control.	Several ML classifiers.	UK Biobank HF cohort; ~1,400 participants.	Best performance for HF detection using XGBoost: accuracy 73.31% (68.33–78.29), AUC 0.837 (0.790–0.879).	NA

According to our recently published study [26], heart failure phenotypes can be noninvasively distinguished using retinal OCT thickness measurements only. Where the unspecified HF phenotype best predicted utilizing both left and right eye measurements, with a accuracy of 67.4% and ROC AUC of 0.692 using the LR model. The analysis here in complements and extends those findings in two ways. First, we target HF vs. normal discrimination using OCT subfields combined with routine clinical variables. Second, we employ SHAP/LIME to link predictions to anatomically specific subfields (e.g., inner macula) and to clinical markers (e.g., HbA1c, BMI), yielding patient level and feature level rationales aligned with HF microvascular and metabolic mechanisms, achieving improved classification performance of accuracy 73.3% and ROC AUC 0.84.

## Interpreting the explainability and clinical importance

Remarkably, several retinal thickness metrics, including macular thickness in the inner/outer subfields consistently exhibit lower values in heart failure patients than normal, with *p*-values typically less than 0.005. Heart failure groups also have thinner central and inner subfield thicknesses, especially the ISOS-RPE layer. Parameters like ISOS-RPE and macular thickness at multiple subfields show significant reductions, reflecting possible retinal structural changes linked to heart failure. In contrast, other retinal measurements, such as ELM-ISOS and INL-ELM thicknesses, show non-significant differences between groups. Global SHAP ranking localized discriminative retinal information to macular subfields that are anatomically plausible for edema/microvascular stress signatures, while clinical covariates (age, gender, BMI, HbA1c, blood pressure, HDL cholesterol) behaved in directionally consistent ways with HF risk biology. At the local (patient) level, LIME profiles enabled healthcare providers audit individual predictions by revealing case-specific drivers (e.g., thicker outer nasal/superior subfields along with elevated BMI and HbA1c) and highlighting instances where model outputs may be influenced by non-causal correlates (e.g., metabolic disease). In this combined clinical/OCT scenario, these complementing tools (SHAP for cohort-level feature attribution and LIME for case-level rationale) are suitable and immediately conform to the transparency criteria for translational machine learning. Given these findings, multimodal retinal imaging may reflect current systemic vascular health in HF patients but can also provide information on the status of HF patients as they undergo treatment, start medications, or acutely decompensate [37].

The retina may exhibit (i) subtle thickening patterns reflecting tissue fluid content; (ii) microvascular rarefaction or dysregulated capillary perfusion; and (iii) neurovascular uncoupling at the macula as a result of the pathophysiology of heart failure [36], which includes neurohormonal activation, venous congestion, interstitial fluid shifts, endothelial dysfunction, and impaired autoregulation [43]. Inner subfields are more prominent in our SHAP ranking, which corresponds to areas where OCT may most clearly show slight variations in capillary density or extracellular water content [44]. These characteristics are probably modulated by systemic metabolic load (HbA1c, BMI) through glycation, oxidative stress, and inflammation, which change glial homeostasis and microvascular integrity [45, 46].

Interestingly, in this study the SHAP analysis revealed a consistent prominence of left-eye macular subfields, particularly the inner temporal, inner nasal, and outer superior regions, among the top discriminative predictors of heart-failure status. While heart failure does not have a known laterality-specific retinal manifestation, several considerations may help contextualize this observation. First, the UK Biobank ophthalmic imaging protocol captures the right eye first followed by the left eye, and prior work has shown that acquisition order can introduce subtle differences related to blinking, tear-film stability, accommodation changes, or participant fatigue, potentially affecting image quality and downstream quantitative measurements [24, 47, 48]. Second, large normative studies have documented physiological inter-ocular asymmetry in macular thickness and retinal layer morphology, with small but consistent differences between left and right eyes reported across healthy populations [49, 50]. These inherent asymmetries may interact with machine-learning models in ways that amplify feature importance on one side. Third, although the present model was not explicitly trained to detect lateralization, the high-dimensional nature of retinal features means that minor distributional differences between eyes may be accentuated by the SHAP ranking process. Finally, our prior study [26]using the same UK Biobank dataset found that left-eye features alone yielded slightly stronger discrimination than right-eye features alone, suggesting that the trend observed here may reflect a reproducible dataset-level characteristic rather than a spurious artifact. Nonetheless, given the absence of a known biological mechanism linking heart-failure pathophysiology to unilateral retinal changes, this left-eye prominence should be interpreted cautiously. Validation in external datasets, along with standardized bilateral imaging protocols, is required to establish whether this lateralization carries physiological relevance or arises from acquisition or sampling variability.

While they demand equipment, expertise, or clinic access, conventional HF diagnostics (ECG, natriuretic peptides, echocardiography, and cardiopulmonary exercise tests) are still considered gold standards [4, 51, 52], OCT-derived retinal features provide structural and microvascular insights linked to HF pathophysiology, by giving mechanism-suggestive indicators that can be monitored over time, aids in clinician validation, and enhances image models or foundation model pipelines. Here, we build on our prior study [26], which demonstrated that OCT-derived retinal features alone carry informative signal related to cardiovascular risk profiles using interpretable models (e.g., SHAP) to localize contribution to specific macular subfields. This has established the feasibility and anatomical interpretability of retina-based cardiology screening. In the present study, we move from risk-factor prediction to HF case control detection and explicitly test whether integrating OCT with routine clinical covariates improves discrimination and clinical utility. Our approach raises accuracy from 67.4% [26]to 73.3%, reflecting a substantial improvement in performance when integrating clinical records with bilateral OCT.

From a clinical perspective, an explainable OCT/clinical screen provides a practical, noninvasively method of identifying patients in primary care or ophthalmology settings who are at a higher risk of heart failure, especially in situations where access to natriuretic peptide testing or echocardiography is limited. In addition to classification, feature-level attributions (e.g., BMI, HbA1c, and macular subfields) place each prediction among risk factors that are associated with guidelines, facilitating collaborative decision making and focused follow up. To determine whether decongestion or treatment optimization is reflected in OCT signatures, the same retinal subfield metrics can be monitored longitudinally alongside symptoms and guideline directed medical therapy (GDMT); model explainability assists clinicians in ensuring that observed changes are physiologic rather than artifact. Finally, transparent explanations enable equity and safety checks: teams can evaluate performance across age, gender, and ethnic subgroups, monitor calibration, and apply decision-curve analysis to ensure the tool adds net clinical benefit prior to implementation.

## Limitations and future work

Although the findings suggest potential associations between OCT-derived retinal characteristics and heart failure, several important limitations must be acknowledged. First, the study is cross-sectional, which limits the ability to infer temporal or causal relationships between retinal OCT features and heart-failure status. The results should therefore be interpreted as hypothesis-generating rather than diagnostic, and longitudinal studies will be necessary to determine whether OCT-derived metrics change with HF progression or treatment response. Second, heart-failure labels were obtained from biobank electronic health records, which may contain a degree of misclassification or incomplete clinical annotation. Also, OCT image quality can be affected by motion artifacts, media opacity, or patient cooperation, and such variability may influence model performance in real-world settings. Although bootstrap and permutation uncertainty estimates, along with stratified cross-validation, yielded consistent performance profiles suggesting an acceptable signal-to-noise ratio, the potential for label noise remains. Third, several ocular and systemic covariates that could influence retinal structure, such as axial length, detailed refractive measures, or contemporaneous hemodynamic parameters, were not uniformly available in the dataset. While key clinical variables such as age, BMI, HbA1c, and blood pressure were included, future studies incorporating complete ocular biometry and more comprehensive clinical panels may improve specificity and help disentangle confounding influences. In addition, the model does not account for medication status or HF treatment history, which could influence both retinal structure and clinical features. Finally, the observed left-eye predominance in feature rankings should be considered exploratory. This pattern may reflect acquisition order effects, physiological inter-eye asymmetry, or sampling variability rather than biological laterality. Validation in independent cohorts and standardized imaging protocols will be required to determine whether this observation has physiological significance or represents dataset-specific variation.

Future work should expand the dataset to include a more diverse global population and encompass a broader range of heart failure types to improve the generalizability of the findings. In addition, prevalence-aware deployment, including calibration to clinic prevalence and task-specific threshold selection (screen, triage, rule-out), prospective, longitudinal studies connecting OCT changes to NT-proBNP, diuretic adjustments, and echocardiographic measures (EF, E/e′), and external validation across scanners, sites, and ancestries with harmonized subfield metrics will be further investigated. In addition, it is recommended to incorporate longitudinal OCT and OCTA follow-up to determine whether retinal structural changes evolve with heart-failure progression or treatment response, thereby validating their potential as temporal or prognostic biomarkers.

## Conclusion

This study demonstrates that integrating retinal OCT subfield measurements with routine clinical indicators within an explainable machine-learning framework can meaningfully differentiate individuals with heart failure from controls. The use of SHAP and LIME enabled transparent interpretation of the learned patterns, consistently identifying inner temporal and nasal macular regions, outer superior subfields, and clinical factors such as age, BMI, and HbA1c as influential contributors, findings that align with established microvascular and metabolic pathways involved in heart failure physiology. These results highlight the potential of retinal OCT as a non-invasive source of structural and microvascular information that can complement traditional clinical assessments, offering an emerging direction for accessible and interpretable cardiovascular risk evaluation. While additional validation is needed before clinical implementation, particularly through longitudinal studies, OCTA integration, and external testing across diverse scanners and populations, the present work provides a foundational step toward leveraging retinal imaging and explainable AI for early heart failure risk stratification. Together, these findings underscore the potential of OCT-based, interpretable models to contribute to scalable, patient-centered approaches for cardiovascular screening and monitoring in both primary-care and ophthalmic settings.

## Electronic supplementary material

Below is the link to the electronic supplementary material.

Supplementary Material 1

## Data Availability

The data analyzed in this study is subject to the following licenses/restrictions: the research described in this study utilized the UK Biobank resource http://www.ukbiobank.ac.uk. However, we cannot publicly share the datasets generated and/or analyzed during this study due to their sensitive nature, potentially compromising research participants’ privacy. Nevertheless, interested parties may access the data upon successful application and approval of the required ethical permissions.

## References

[1] Alsindi F, Manla Y, Soliman M, Hamour IM, Ghalib H, Bader F. Heart failure clinic No-show rates and effect on heart failure hospitalizations: a real-world experience from the Middle East. J Card Fail. 2022, Apr;28:S115–16. 10.1016/j.cardfail.2022.03.296.

[2] Tomasoni D, et al. The role of multimorbidity in patients with heart failure across the left ventricular ejection fraction spectrum: data from the Swedish heart failure registry. Eur J Heart Fail. 2024, Apr;26:854–68. 10.1002/ejhf.3112. 38131248 10.1002/ejhf.3112

[3] Ben-Assuli O, Heart T, Klempfner R, Padman R. Human-machine collaboration for feature selection and integration to improve congestive heart failure risk prediction. Decis Support Syst. 2023, Sep;172. 10.1016/j.dss.2023.113982.

[4] Younis SMA, Hadjileontiadis LJ, Stefanini C, Khandoker AH. Non-invasive technologies for heart failure, systolic and diastolic dysfunction modeling: a scoping review,” 2023. Front Media SA. 10.3389/fbioe.2023.1261022. 10.3389/fbioe.2023.1261022 PMC10619666 37920244

[5] Nakao YM, et al. Prognosis, characteristics, and provision of care for patients with the unspecified heart failure electronic health record phenotype: a population-based linked cohort study of 95262 individuals. EClinicalMedicine. 2023, Sep;63:102164. 10.1016/j.eclinm.2023.102164. 37662516 10.1016/j.eclinm.2023.102164 PMC10474358

[6] Docherty KF, et al. Heart failure diagnosis in the general community - who, how and when? A clinical consensus statement of the heart failure Association (HFA) of the European Society of Cardiology (ESC). Eur J Heart Fail. 2023, Aug;25:1185–98. 10.1002/ejhf.2946. 37368511 10.1002/ejhf.2946

[7] Castiglione V, Aimo A, Vergaro G, Saccaro L, Passino C, Emdin M. Biomarkers for the diagnosis and management of heart failure. Springer. 2022, Mar. 10.1007/s10741-021-10105-w. 10.1007/s10741-021-10105-w PMC8898236 33852110

[8] Inamdar AA, Inamdar AC. Heart failure: diagnosis, management and utilization. MDPI. 2016, Jul. 10.3390/jcm5070062. 10.3390/jcm5070062 PMC4961993 27367736

[9] Sultan RH, et al. Correlations between kidney and heart function bioindicators and the expressions of Toll-like, ACE2, and NRP-1 receptors in COVID-19. Vaccines (Basel). 2022, Jul;10. 10.3390/vaccines10071106. 10.3390/vaccines10071106 PMC9319872 35891270

[10] Widatalla N, Younis SA, Khandoker A. Heart rate transition patterns reveal autonomic dysfunction in heart failure with renal function decline: a symbolic and Markov model approach. Biodata Min. 2025, Dec;18. 10.1186/s13040-025-00460-x. 10.1186/s13040-025-00460-x PMC12180264 40542415

[11] Widatalla N, Younis SA, Ghosh SK, Khandoker A. Symbolic heart rate transition motifs during nocturnal sleep are associated with diabetic complications in type 2 diabetes. PLoS One. 2025, Sep;20. 10.1371/journal.pone.0333067. 10.1371/journal.pone.0333067 PMC12459800 40991644

[12] Younis SMA, Jelinek HF, Almahmeed WA, Khalaf K. Quantitative gait Analytics reveal atrial fibrillation-specific patterns in balance and mobility: implications for Fall prevention. IEEE Access. 2025, Nov;13:196698–712. 10.1109/ACCESS.2025.3633446.

[13] Elendu C, et al. A comprehensive review of heart failure: unraveling the etiology, decoding pathophysiological mechanisms, navigating diagnostic modalities, exploring pharmacological interventions, advocating lifestyle modifications, and charting the horizon of emerging therapies in the complex landscape of chronic cardiac dysfunction. Lippincott Williams Wilkins. 2024, Jan. 10.1097/MD.0000000000036895. 10.1097/MD.0000000000036895 PMC10798706 38241566

[14] Gutterman DD, et al. The Human microcirculation: regulation of flow and beyond. Lippincott Williams Wilkins. 2016, Jan. 10.1161/CIRCRESAHA.115.305364. 10.1161/CIRCRESAHA.115.305364 PMC4742348 26837746

[15] Eijgen JV, et al. Retinal vessel analysis to assess microvascular function in the healthy eye: a systematic review on the response to acute physiological and pathological stressors. Elsevier Inc. 2025, Mar. 10.1016/j.survophthal.2024.11.008. 10.1016/j.survophthal.2024.11.008 39592075

[16] Al-Absi HRH, Islam MT, Refaee MA, Chowdhury MEH, Alam T. Cardiovascular disease diagnosis from DXA scan and retinal images using deep learning. Sensors. 2022, Jun;22. 10.3390/s22124310. 10.3390/s22124310 PMC9228833 35746092

[17] Hardie R, Erskine JG, Gozzard DI. Management of infection in the neutropenic patient. 1986. 10.1136/bmj.293.6550.817-a. 10.1136/bmj.293.6547.627-a PMC1341412 3092957

[18] Vaghefi E, et al. Development and validation of a deep-learning model to predict 10-year atherosclerotic cardiovascular disease risk from retinal images using the UK Biobank and EyePACS 10K datasets. Cardiovasc Digit Health J. 2024, Apr;5:59–69. 10.1016/j.cvdhj.2023.12.004. 38765618 10.1016/j.cvdhj.2023.12.004 PMC11096659

[19] Tapp RJ, et al. Retinal microvascular associations with cardiometabolic risk factors differ by diabetes status: results from the UK Biobank. Diabetologia. 2022, Oct;65:1652–63. 10.1007/s00125-022-05745-y. 35852586 10.1007/s00125-022-05745-y PMC9477904

[20] Weerts J, et al. Retinal vascular changes in heart failure with preserved ejection fraction using optical coherence tomography angiography. J Clin Med. 2024, Apr;13. 10.3390/jcm13071892. 10.3390/jcm13071892 PMC11012357 38610657

[21] Collins R. UK biobank: protocol for a large-scale prospective epidemiological resource. https://scholar.google.com/scholar?hl=en&as_sdt=0%2C5&q=Collins%2C+R.+%282007%29.+UK+biobank%3A+protocol+for+a+large-scale+prospective+epidemiological+resource.&btnG=.

[22] Sudlow C, et al. UK Biobank: an open access resource for identifying the causes of a wide range of complex Diseases of Middle and old age. PLoS Med. 2015, Mar;12. 10.1371/journal.pmed.1001779. 10.1371/journal.pmed.1001779 PMC4380465 25826379

[23] Allen N, et al. UK Biobank: current status and what it means for epidemiology. Health Policy Technol. 2012, Sep;1:123–26. 10.1016/j.hlpt.2012.07.003.

[24] Keane PA, et al. Optical coherence tomography in the UK Biobank study - rapid automated analysis of retinal thickness for large population-based studies. PLoS One. 2016, Oct;11. 10.1371/journal.pone.0164095. 10.1371/journal.pone.0164095 PMC5055325 27716837

[25] Ko F, et al. Associations with retinal Pigment Epithelium thickness measures in a large cohort: results from the UK Biobank. In: Ophthalmology. Elsevier Inc; 2017 Jan. p. 105–17. 10.1016/j.ophtha.2016.07.033. 10.1016/j.ophtha.2016.07.033 27720551

[26] Younis SMA, Ghosh SK, Raja H, Alskafi FA, Yousefi S, Khandoker AH. Prediction of heart failure risk factors from retinal optical imaging via explainable machine learning. Front Med (Lausanne). 2025;12. 10.3389/fmed.2025.1551557. 10.3389/fmed.2025.1551557 PMC11955505 40166058

[27] Wang H, Liu W, Cai W, Lu Y, Wan C. Efficient attacks on strong PUFs via Covariance and boolean modeling. ACM Transact Des Autom Electron Syst. 2024, Sep;29. 10.1145/3687469.

[28] Diaz GI, Fokoue-Nkoutche A, Nannicini G, Samulowitz H. An effective algorithm for hyperparameter optimization of neural networks. IBM J Res Dev. 2017, Jul;61. 10.1147/JRD.2017.2709578.

[29] Yang L, et al. Study of cardiovascular disease prediction model based on random forest in eastern China. Sci Rep. 2020, Dec;10. 10.1038/s41598-020-62133-5. 10.1038/s41598-020-62133-5 PMC7090086 32251324

[30] G A, Ganesh B, Ganesh A, Srinivas C, Dhanraj, Mensinkal K. Logistic regression technique for prediction of cardiovascular disease. Global Transitions Proc. 2022, Jun;3:127–30. 10.1016/j.gltp.2022.04.008.

[31] Wang Y, et al. Clinical prediction of heart failure in hemodialysis patients: based on the Extreme Gradient Boosting method. Front Genet. 2022, Apr;13. 10.3389/fgene.2022.889378. 10.3389/fgene.2022.889378 PMC9086166 35559036

[32] Cawley GC, Talbot NLC. On over-fitting in model selection and subsequent selection bias in performance evaluation. 2010.

[33] Younis SMA, et al. Prediction of heart failure patients with distinct left ventricular ejection fraction levels using circadian ECG features and machine learning. PLoS One. 2024, May;19. 10.1371/journal.pone.0302639. 10.1371/journal.pone.0302639 PMC11090346 38739639

[34] Lundberg SM, et al. From local explanations to global understanding with explainable AI for trees. Nat Mach Intell. 2020, Jan;2:56–67. 10.1038/s42256-019-0138-9. 32607472 10.1038/s42256-019-0138-9 PMC7326367

[35] Ribeiro MT, Singh S, Guestrin C. ‘Why should i trust you?’ explaining the predictions of any classifier. Proceedings of the ACM SIGKDD International Conference on Knowledge Discovery and Data Mining, Association for Computing Machinery. 2016 Aug, pp. 1135–44. doi: 10.1145/2939672.2939778.

[36] Rakusiewicz K, Kanigowska K, Hautz W, Ziółkowska L. The impact of chronic heart failure on retinal vessel density assessed by optical coherence tomography angiography in children with dilated cardiomyopathy. J Clin Med. 2021, Jun;10. 10.3390/jcm10122659. 10.3390/jcm10122659 PMC8235508 34208770

[37] Bisen JB, Sikora H, Aneja A, Shah SJ, Mirza RG. Retinal imaging as a window into cardiovascular health: towards harnessing retinal Analytics for Precision cardiovascular Medicine. In: Jun. *Multidisciplinary Digital Publishing Institute (MDPI)*; 2025. 10.3390/jcdd12060230. 10.3390/jcdd12060230 PMC12194434 40558665

[38] Kamalzadeh H, Khorrami F, Ahmadi A, Mirlohi SR, Vatankhah M, Choobin N. Through the eye to the heart: a scoping review of artificial intelligence in retinal imaging for cardiovascular disease assessment. BMC Med Inf Decis Mak. 2025, Nov. 10.1186/s12911-025-03300-4. 10.1186/s12911-025-03300-4 PMC12750919 41299604

[39] Hou Q, et al. Is an ultra large natural image-based foundation model superior to a retina-specific model for detecting ocular and systemic Diseases? 2025, Sep. 10.1016/j.xops.2025.100923 PMC12548085 41140901

[40] Tseng RMWW, et al. Validation of a deep-learning-based retinal biomarker (Reti-CVD) in the prediction of cardiovascular disease: data from UK Biobank. BMC Med. 2023, Dec;21. 10.1186/s12916-022-02684-8. 10.1186/s12916-022-02684-8 PMC9872417 36691041

[41] S B, Kadry S, Dhanaraj RK, et al. Res-unet based blood vessel segmentation and cardio vascular disease prediction using chronological chef-based optimization algorithm based deep residual network from retinal fundus images. Multimed Tools Appl. 2024;83:87929–58. https://doi-org.khalifa.idm.oclc.org/10.1007/s11042-024-18810-y.

[42] Zhou Y, et al. A foundation model for generalizable disease detection from retinal images. Nature. 2023, Oct;622:156–63. 10.1038/s41586-023-06555-x. 37704728 10.1038/s41586-023-06555-x PMC10550819

[43] La Vecchia G, Fumarulo I, Caffè A, Chiatto M, Montone RA, Aspromonte N. Microvascular dysfunction across the spectrum of heart failure pathology: pathophysiology, clinical features and therapeutic Implications. *Multidisciplinary Digital Publishing Institute (MDPI)*; 2024 Jul. 10.3390/ijms25147628. 10.3390/ijms25147628 PMC11277452 39062871

[44] Haydinger CD, Ferreira LB, Williams KA, Smith JR. Mechanisms of macular edema. Front Media S.A. 2023. 10.3389/fmed.2023.1128811. 10.3389/fmed.2023.1128811 PMC10027768 36960343

[45] Chua SYL, et al. Associations between HbA1c across the normal range, diagnosed, and undiagnosed diabetes and retinal layer thickness in UK Biobank cohort. Transl Vis Sci Technol. 2023;12. 10.1167/tvst.12.2.25. 10.1167/tvst.12.2.25 PMC9940769 36795065

[46] Patel PJ, et al. Spectral-domain optical coherence tomography imaging in 67 321 adults: associations with macular thickness in the UK biobank study. Ophthalmology. 2016, Apr;123:829–40. 10.1016/j.ophtha.2015.11.009. 26746598 10.1016/j.ophtha.2015.11.009

[47] Napoli PE, et al. Fourier-domain OCT imaging of the ocular surface and tear film dynamics: a review of the state of the art and an integrative model of the tear behavior during the inter-blink period and visual fixation. MDPI. 2020, Mar. 10.3390/jcm9030668. 10.3390/jcm9030668 PMC7141198 32131486

[48] Lin K, et al. Comparison of the repeatability and reproducibility of corneal thickness mapping using optical coherence tomography according to tear film break-up time. BMC Ophthalmol. 2024, Dec;24. 10.1186/s12886-024-03536-0. 10.1186/s12886-024-03536-0 PMC11227131 38970043

[49] Muñoz-Gallego A, et al. Interobserver reproducibility and interocular symmetry of the macular ganglion cell complex: assessment in healthy children using optical coherence tomography. BMC Ophthalmol. 2020, May;20. 10.1186/s12886-020-01379-z. 10.1186/s12886-020-01379-z PMC7245936 32448232

[50] Wagner SK, et al. Associations between unilateral amblyopia in childhood and cardiometabolic disorders in adult life: a cross-sectional and longitudinal analysis of the UK Biobank. EClinicalMedicine. 2024, Apr;70. 10.1016/j.eclinm.2024.102493. 10.1016/j.eclinm.2024.102493 PMC11056416 38685932

[51] Alyounis S, Khandoker A, Stefanini C, Hadjileontiadis LJ. A hybrid CNN-LSTM model for heart failure detection using raw ECG signals. 2024 Computing in Cardiology Conference (CinC), Computing in Cardiology. 2024 Dec. 10.22489/cinc.2024.197.

[52] Alyounis S, Khandoker A, Stefanini C, Hadjileontiadis L. Assessment of Serum creatinine and Serum sodium prognostic potential in heart failure patients using machine learning. Proceedings of the Annual International Conference of the IEEE Engineering in Medicine and Biology Society, EMBS. Institute of Electrical and Electronics Engineers Inc; 2024. 10.1109/EMBC53108.2024.10782107. 10.1109/EMBC53108.2024.10782107 40039648

