# Supplementary material for: From retina to heart: explainable machine learning using OCT and Clinical covariates for heart failure screening
Source: BioData Min. 2026 Feb 28;19:28. doi: 10.1186/s13040-026-00529-1 (PMC13097962; doi:10.1186/s13040-026-00529-1)
Supplement: Supplementary file 1 — Supplementary Material 1 [file 13040_2026_529_MOESM1_ESM.docx]

Supplementary Material

# Supplement Table S1: Derived retinal optical coherence tomography features and their definitions

| **Category** | **Measurement** | **Physical Meaning** |
| --- | --- | --- |
| ELM-ISOS Thickness (Left/Right) | ELM-ISOS Average Thickness | Measures the thickness between the External Limiting Membrane (ELM) and Inner Segment-Outer Segment (ISOS) junction, critical for photoreceptor function. |
|  | ELM-ISOS Central Subfield | Represents the thickness of the ELM-ISOS junction specifically in the central subfield of the macula, related to central vision. |
|  | ELM-ISOS Inner Subfield | Measures the thickness of the ELM-ISOS junction in the inner subfield of the retina, surrounding the central subfield. |
|  | ELM-ISOS Outer Subfield | Captures the thickness of the ELM-ISOS junction in the outer subfield, further away from the central vision area. |
| INL-ELM Thickness (Left/Right) | INL-ELM Average Thickness | The thickness between the Inner Nuclear Layer (INL) and External Limiting Membrane (ELM), indicating the integrity of the retina's inner layers. |
|  | INL-ELM Central Subfield | Thickness between the INL and ELM in the central subfield, key for evaluating retinal layer function in central vision. |
|  | INL-ELM Inner Subfield | INL-ELM thickness in the inner region surrounding the central subfield. |
|  | INL-ELM Outer Subfield | INL-ELM thickness in the outer subfield of the macula. |
| INL-RPE Thickness (Left/Right) | INL-RPE Average Thickness | Measures the thickness between the Inner Nuclear Layer (INL) and Retinal Pigment Epithelium (RPE), important for evaluating retinal integrity. |
|  | INL-RPE Central Subfield | The INL-RPE thickness in the central macular region, linked to core visual function. |
|  | INL-RPE Inner Subfield | Thickness of the INL-RPE junction in the inner macular subfield. |
|  | INL-RPE Outer Subfield | INL-RPE thickness in the outer subfield, extending away from the fovea. |
| ISOS-RPE Thickness (Left/Right) | ISOS-RPE Average Thickness | Thickness between the Inner Segment-Outer Segment (ISOS) junction and the Retinal Pigment Epithelium (RPE), critical for assessing photoreceptor health. |
|  | ISOS-RPE Central Subfield | ISOS-RPE thickness in the central subfield of the retina is linked to visual acuity and central photoreceptor function. |
|  | ISOS-RPE Inner Subfield | Thickness of the ISOS-RPE junction in the inner subfield of the retina. |
|  | ISOS-RPE Outer Subfield | ISOS-RPE thickness in the outer subfield, representing peripheral photoreceptor function. |
| Macular Thickness | Macular Thickness at Central Subfield (Left/Right) | Central macular thickness is a key measurement in assessing macular health, particularly for central vision. |
|  | Macular Thickness at Inner Subfields (Inferior, Nasal, Superior, Temporal - Left/Right) | Thickness in various inner subfields of the macula is related to the health and function of areas surrounding the central macula. |
|  | Macular Thickness at Outer Subfields (Inferior, Nasal, Superior, Temporal - Left/Right) | Thickness in the outer macular subfields, extending further from the central vision area, is important for peripheral retinal health. |
|  | Overall Macular Thickness (Left/Right) | The average thickness across the entire macula gives an overall measure of macular health. |
| Intraocular Pressure (IOP) (Left/Right) | IOP Corneal Compensated | Measures intraocular pressure, adjusted for corneal thickness, important for assessing glaucoma risk. |
|  | IOP Goldmann Correlated | The Goldmann method measures intraocular pressure, the gold standard for glaucoma diagnosis. |

# Supplement Table S2: The demographic and clinical characteristics of Normal and Heart Failure groups.

| **Medical Variables** | **Overall**  **(1402)** | **Normal (701)** | **HF**  **(701)** | **p-value^*^** |
| --- | --- | --- | --- | --- |
| Gender (M/F) | 752/650 | 287/414 | 465/236 | **<0.001** |
| Age (yrs.) | 59.4±7.9 | 56.2 ± 8.0 | 62.6 ± 6.1 | **<0.001** |
| Smoking (Yes/No) | 722/680 | 308/393 | 414/287 | **<0.001** |
| Alcohol (Yes/No) | 1317/85 | 675/26 | 642/59 | **<0.001** |
| BMI (kg/m^2^) | 28.4 ± 5.4 | 26.9 ± 4.9 | 29.9 ± 5.3 | **<0.001** |
| HDL (mmol/L) | 1.4 ± 0.4 | 1.52 ± 0.39 | 1.3 ± 0.3 | **<0.001** |
| Glucose (mmol/L) | 5.4 ± 1.6 | 5.2 ± 0.9 | 5.7 ± 2.0 | **<0.001** |
| HbA1c (%) | 38.1 ± 9.5 | 35.8 ± 6.5 | 40.4 ± 11.3 | **<0.001** |
| SBP (mmHg) | 141.5 ± 20 | 138.1 ± 19.4 | 144.9 ± 20.5 | **<0.001** |
| DBP (mmHg) | 81.4 ± 11.1 | 81.4 ± 10.5 | 81.4 ± 11.6 | 0.564 |

** Variables represented in (Mean±Std), BMI, Body mass index, HDL, HDL cholesterol, HbA1c, HbA1c Glycated hemoglobin, SBP, Systolic blood pressure, DBP,* *Diastolic blood pressure. Significant p values are represented in bold*

**Supplement Table S3: Retinal optical coherence tomography measurements for left eye**

| Feature | Normal (701) | HF (701) | Overall (1402) | P-value |
| --- | --- | --- | --- | --- |
| ELM-ISOS thickness | 24.10 ± 3.01 | 24.10 ± 3.64 | 24.10 ± 3.34 | 9.8705e-01 |
| INL-ELM thickness | 80.00 ± 8.68 | 80.34 ± 10.82 | 80.17 ± 9.81 | 5.1083e-01 |
| INL-RPE thickness | 141.63 ± 12.88 | 140.77 ± 16.37 | 141.20 ± 14.73 | 2.7475e-01 |
| ISOS-RPE thickness | 37.52 ± 5.59 | 36.32 ± 6.78 | 36.92 ± 6.24 | **3.0405e-04** |
| ELM-ISOS thickness of central subfield | 28.25 ± 3.93 | 27.74 ± 4.06 | 28.00 ± 4.00 | **1.5449e-02** |
| ELM-ISOS thickness of inner subfield | 24.87 ± 3.09 | 24.72 ± 3.64 | 24.80 ± 3.37 | 4.1738e-01 |
| ELM-ISOS thickness of outer subfield | 23.72 ± 3.13 | 23.78 ± 3.74 | 23.75 ± 3.45 | 7.4924e-01 |
| INL-ELM thickness of the central subfield | 105.96 ± 14.77 | 105.22 ± 17.09 | 105.59 ± 15.97 | 3.8759e-01 |
| INL-ELM thickness of the inner subfield | 92.78 ± 10.56 | 92.72 ± 12.76 | 92.75 ± 11.71 | 9.1555e-01 |
| INL-ELM thickness of the outer subfield | 75.25 ± 8.40 | 75.76 ± 10.58 | 75.50 ± 9.55 | 3.2089e-01 |
| INL-RPE thickness of central subfield | 175.50 ± 21.09 | 172.66 ± 23.96 | 174.08 ± 22.60 | **1.8945e-02** |
| INL-RPE thickness of inner subfield | 155.60 ± 15.19 | 154.43 ± 18.50 | 155.01 ± 16.93 | 1.9542e-01 |
| INL-RPE thickness of outer subfield | 136.23 ± 12.49 | 135.54 ± 15.92 | 135.89 ± 14.31 | 3.6437e-01 |
| ISOS-RPE thickness of central subfield | 41.29 ± 8.71 | 39.71 ± 8.96 | 40.50 ± 8.87 | **8.5570e-04** |
| ISOS-RPE thickness of inner subfield | 37.95 ± 6.55 | 36.99 ± 7.41 | 37.47 ± 7.01 | **1.0361e-02** |
| ISOS-RPE thickness of outer subfield | 37.26 ± 5.43 | 36.00 ± 6.71 | 36.63 ± 6.13 | **1.1691e-04** |
| Macular thickness at the central subfield | 266.01 ± 31.35 | 267.10 ± 36.45 | 266.56 ± 33.99 | 5.4842e-01 |
| Macular thickness at the inner inferior subfield | 307.32 ± 24.67 | 300.05 ± 31.53 | 303.69 ± 28.53 | **1.7050e-06** |
| Macular thickness at the inner nasal subfield | 313.90 ± 28.60 | 308.12 ± 32.12 | 311.01 ± 30.54 | **3.8355e-04** |
| Macular thickness at the inner superior subfield | 307.52 ± 29.66 | 299.39 ± 35.48 | 303.46 ± 32.94 | **3.5151e-06** |
| Macular thickness at the inner temporal subfield | 297.07 ± 29.55 | 289.68 ± 35.12 | 293.38 ± 32.65 | **2.1791e-05** |
| Macular thickness at the outer inferior subfield | 263.50 ± 22.48 | 258.19 ± 27.74 | 260.85 ± 25.38 | **8.6384e-05** |
| Macular thickness at the outer nasal subfield | 287.37 ± 22.36 | 282.30 ± 27.13 | 284.83 ± 24.98 | **1.4141e-04** |
| Macular thickness at the outer superior subfield | 264.28 ± 22.77 | 258.34 ± 26.92 | 261.31 ± 25.10 | **8.8712e-06** |
| Macular thickness at the outer temporal subfield | 248.88 ± 21.45 | 243.86 ± 28.20 | 246.37 ± 25.17 | **1.8248e-04** |
| Overall macular thickness | 275.00 ± 19.81 | 269.45 ± 24.70 | 272.22 ± 22.55 | **3.9193e-06** |
| IOP Corneal compensated | 15.95 ± 4.64 | 15.84 ± 3.97 | 15.90 ± 4.32 | 6.3024e-01 |
| IOP Goldmann correlated | 15.90 ± 4.77 | 15.60 ± 3.94 | 15.75 ± 4.38 | 2.0185e-01 |

*Significant p values are represented in bold*

**Supplement Table S4: Retinal optical coherence tomography measurements for right eye**

| Feature | Normal (701) | HF (701) | Overall (1402) | P-value |
| --- | --- | --- | --- | --- |
| ELM-ISOS thickness | 24.04 ± 3.33 | 24.21 ± 3.50 | 24.13 ± 3.41 | 3.5470e-01 |
| INL-ELM thickness | 79.78 ± 9.60 | 80.41 ± 9.81 | 80.10 ± 9.71 | 2.2558e-01 |
| INL-RPE thickness | 141.13 ± 14.44 | 140.96 ± 14.29 | 141.05 ± 14.36 | 8.2111e-01 |
| ISOS-RPE thickness | 37.31 ± 6.26 | 36.34 ± 6.85 | 36.82 ± 6.58 | **5.6909e-03** |
| ELM-ISOS thickness of central subfield | 28.39 ± 3.75 | 28.13 ± 4.20 | 28.26 ± 3.98 | 2.2660e-01 |
| ELM-ISOS thickness of inner subfield | 24.84 ± 3.31 | 24.87 ± 3.52 | 24.86 ± 3.42 | 8.5153e-01 |
| ELM-ISOS thickness of outer subfield | 23.64 ± 3.44 | 23.87 ± 3.61 | 23.76 ± 3.53 | 2.3350e-01 |
| INL-ELM thickness of the central subfield | 106.36 ± 14.45 | 106.99 ± 15.92 | 106.67 ± 15.20 | 4.3566e-01 |
| INL-ELM thickness of the inner subfield | 92.60 ± 11.17 | 93.20 ± 11.54 | 92.90 ± 11.36 | 3.2168e-01 |
| INL-ELM thickness of the outer subfield | 75.00 ± 9.38 | 75.64 ± 9.64 | 75.32 ± 9.51 | 2.1064e-01 |
| INL-RPE thickness of central subfield | 176.64 ± 21.01 | 175.37 ± 21.80 | 176.00 ± 21.41 | 2.6777e-01 |
| INL-RPE thickness of inner subfield | 155.36 ± 16.53 | 155.18 ± 16.41 | 155.27 ± 16.46 | 8.3698e-01 |
| INL-RPE thickness of outer subfield | 135.60 ± 13.95 | 135.47 ± 13.92 | 135.54 ± 13.93 | 8.6080e-01 |
| ISOS-RPE thickness of central subfield | 41.89 ± 8.81 | 40.25 ± 9.29 | 41.07 ± 9.09 | **6.9593e-04** |
| ISOS-RPE thickness of inner subfield | 37.92 ± 7.07 | 37.10 ± 7.48 | 37.51 ± 7.29 | **3.5918e-02** |
| ISOS-RPE thickness of outer subfield | 36.96 ± 6.10 | 35.97 ± 6.74 | 36.46 ± 6.45 | **3.9606e-03** |
| Macular thickness at the central subfield | 268.23 ± 31.69 | 271.25 ± 33.25 | 269.74 ± 32.51 | 8.2782e-02 |
| Macular thickness at the inner inferior subfield | 309.00 ± 27.47 | 305.64 ± 32.60 | 307.32 ± 30.18 | **3.6995e-02** |
| Macular thickness at the inner nasal subfield | 315.09 ± 26.69 | 310.19 ± 29.92 | 312.64 ± 28.45 | **1.2412e-03** |
| Macular thickness at the inner superior subfield | 311.19 ± 30.63 | 304.74 ± 33.11 | 307.97 ± 32.04 | **1.5651e-04** |
| Macular thickness at the inner temporal subfield | 301.37 ± 26.65 | 297.66 ± 27.68 | 299.52 ± 27.22 | **1.0696e-02** |
| Macular thickness at the outer inferior subfield | 263.09 ± 24.30 | 261.46 ± 35.10 | 262.27 ± 30.19 | 3.1180e-01 |
| Macular thickness at the outer nasal subfield | 283.10 ± 22.13 | 278.64 ± 28.27 | 280.87 ± 25.48 | **1.0456e-03** |
| Macular thickness at the outer superior subfield | 269.34 ± 22.96 | 264.90 ± 27.69 | 267.12 ± 25.52 | **1.1139e-03** |
| Macular thickness at the outer temporal subfield | 258.97 ± 22.95 | 256.94 ± 24.04 | 257.96 ± 23.52 | 1.0544e-01 |
| Overall macular thickness | 277.62 ± 20.82 | 274.31 ± 25.27 | 275.97 ± 23.20 | **7.5715e-03** |
| IOP Corneal compensated | 16.04 ± 4.24 | 16.02 ± 4.21 | 16.03 ± 4.23 | 9.6358e-01 |
| IOP Goldmann correlated | 15.90 ± 4.08 | 15.88 ± 4.41 | 15.89 ± 4.25 | 9.3235e-01 |

*Significant p values are represented in bold*

**Supplement Table S5: A summary table listing all variables used in the classification models**

| **Variable Name** | **Unit** | **Missing data%** | | **Imputation Strategy** |
| --- | --- | --- | --- | --- |
|  |  | **Normal** | **HF** |  |
| **Medical Variables** | | | | |
| Gender | Male/Female | 0 | 0 | NA |
| Age | Years | 0 | 0 | NA |
| Smoking | Yes/No | 0 | 2 | Not applicable (categorical encoding) |
| Alcohol | Yes/No | 1 | 0 | Not applicable (categorical encoding) |
| BMI | kg/m^2^ | 0 | 0 | NA |
| HDL | mmol/L | 2 | 0 | Median |
| Glucose | mmol/L | 0 | 1 | Median |
| HbA1c | % | 0 | 0 | NA |
| SBP | mmHg | 1 | 0 | Median |
| DBP | mmHg | 1 | 0 | Median |
| **Retinal optical coherence tomography measurements for Left eye** | | | | |
| ELM-ISOS thickness | µm | 0 | 0 | NA |
| INL-ELM thickness | µm | 0 | 0 | NA |
| INL-RPE thickness | µm | 0 | 1 | Median |
| ISOS-RPE thickness | µm | 0 | 0 | NA |
| ELM-ISOS thickness of central subfield | µm | 3 | 0 | Median |
| ELM-ISOS thickness of inner subfield | µm | 0 | 0 | NA |
| ELM-ISOS thickness of outer subfield | µm | 0 | 0 | NA |
| INL-ELM thickness of the central subfield | µm | 0 | 0 | NA |
| INL-ELM thickness of the inner subfield | µm | 0 | 0 | NA |
| INL-ELM thickness of the outer subfield | µm | 0 | 0 | NA |
| INL-RPE thickness of central subfield | µm | 0 | 2 | Median |
| INL-RPE thickness of inner subfield | µm | 0 | 0 | NA |
| INL-RPE thickness of outer subfield | µm | 0 | 0 | NA |
| ISOS-RPE thickness of central subfield | µm | 1 | 0 | Median |
| ISOS-RPE thickness of inner subfield | µm | 0 | 0 | NA |
| ISOS-RPE thickness of outer subfield | µm | 0 | 0 | NA |
| Macular thickness at the central subfield | µm | 0 | 0 | NA |
| Macular thickness at the inner inferior subfield | µm | 1 | 1 | Median |
| Macular thickness at the inner nasal subfield | µm | 0 | 0 | NA |
| Macular thickness at the inner superior subfield | µm | 0 | 0 | NA |
| Macular thickness at the inner temporal subfield | µm | 0 | 0 | NA |
| Macular thickness at the outer inferior subfield | µm | 0 | 0 | NA |
| Macular thickness at the outer nasal subfield | µm | 0 | 0 | NA |
| Macular thickness at the outer superior subfield | µm | 0 | 0 | NA |
| Macular thickness at the outer temporal subfield | µm | 0 | 0 | NA |
| Overall macular thickness | µm | 1 | 1 | Median |
| IOP Corneal compensated | mmHg | 0 | 0 | NA |
| IOP Goldmann correlated | mmHg | 3 | 0 | Median |
| **Retinal optical coherence tomography measurements for right eye** | | | | |
| ELM-ISOS thickness | µm | 0 | 0 | NA |
| INL-ELM thickness | µm | 0 | 0 | NA |
| INL-RPE thickness | µm | 0 | 0 | NA |
| ISOS-RPE thickness | µm | 0 | 0 | NA |
| ELM-ISOS thickness of central subfield | µm | 0 | 0 | NA |
| ELM-ISOS thickness of inner subfield | µm | 0 | 3 | Median |
| ELM-ISOS thickness of outer subfield | µm | 0 | 0 | NA |
| INL-ELM thickness of the central subfield | µm | 0 | 0 | NA |
| INL-ELM thickness of the inner subfield | µm | 1 | 0 | Median |
| INL-ELM thickness of the outer subfield | µm | 0 | 0 | NA |
| INL-RPE thickness of central subfield | µm | 0 | 0 | NA |
| INL-RPE thickness of inner subfield | µm | 0 | 0 | NA |
| INL-RPE thickness of outer subfield | µm | 0 | 0 | NA |
| ISOS-RPE thickness of central subfield | µm | 0 | 0 | NA |
| ISOS-RPE thickness of inner subfield | µm | 0 | 0 | NA |
| ISOS-RPE thickness of outer subfield | µm | 0 | 2 | Median |
| Macular thickness at the central subfield | µm | 0 | 0 | NA |
| Macular thickness at the inner inferior subfield | µm | 0 | 0 | NA |
| Macular thickness at the inner nasal subfield | µm | 0 | 0 | NA |
| Macular thickness at the inner superior subfield | µm | 2 | 0 | Median |
| Macular thickness at the inner temporal subfield | µm | 0 | 0 | NA |
| Macular thickness at the outer inferior subfield | µm | 0 | 0 | NA |
| Macular thickness at the outer nasal subfield | µm | 0 | 0 | NA |
| Macular thickness at the outer superior subfield | µm | 0 | 0 | NA |
| Macular thickness at the outer temporal subfield | µm | 0 | 1 | Median |
| Overall macular thickness | µm | 2 | 1 | Median |
| IOP Corneal compensated | mmHg | 0 | 0 | NA |
| IOP Goldmann correlated | mmHg | 2 | 0 | Median |
